# Holistic understanding of trimethoprim resistance in *Streptococcus pneumoniae* using an integrative approach of genome-wide association study, resistance reconstruction, and machine learning

**DOI:** 10.1128/mbio.01360-24

**Published:** 2024-08-09

**Authors:** Nguyen-Phuong Pham, Hélène Gingras, Chantal Godin, Jie Feng, Alexis Groppi, Macha Nikolski, Philippe Leprohon, Marc Ouellette

**Affiliations:** 1 Centre de Recherche en Infectiologie du Centre de Recherche du CHU de Québec and Département de Microbiologie, Infectiologie et Immunologie, Faculté de Médecine, Université Laval, Québec City, Québec, Canada; 2 State Key Laboratory of Microbial Resources, Institute of Microbiology, Chinese Academy of Sciences, Beijing, China; 3 Bordeaux Bioinformatics Center and CNRS, Institut de Biochimie et Génétique Cellulaires (IBGC) UMR 5095, Université de Bordeaux, Bordeaux, France; McMaster University, Hamilton, Ontario, Canada

**Keywords:** *Streptococcus pneumoniae*, trimethoprim, drug resistance mechanisms, genome-wide association study, machine learning

## Abstract

Antimicrobial resistance (AMR) is a public health threat worldwide. Next-generation sequencing (NGS) has opened unprecedented opportunities to accelerate AMR mechanism discovery and diagnostics. Here, we present an integrative approach to investigate trimethoprim (TMP) resistance in the key pathogen *Streptococcus pneumoniae*. We explored a collection of 662 *S*. *pneumoniae* genomes by conducting a genome-wide association study (GWAS), followed by functional validation using resistance reconstruction experiments, combined with machine learning (ML) approaches to predict TMP minimum inhibitory concentration (MIC). Our study showed that multiple additive mutations in the *folA* and *sulA* loci are responsible for TMP non-susceptibility in *S. pneumoniae* and can be used as key features to build ML models for digital MIC prediction, reaching an average accuracy within ±1 twofold dilution factor of 86.3%. Our roadmap of *in silico* analysis—wet-lab validation—diagnostic tool building could be adapted to explore AMR in other combinations of bacteria–antibiotic.

**IMPORTANCE:**

In the age of next-generation sequencing (NGS), while data-driven methods such as genome-wide association study (GWAS) and machine learning (ML) excel at finding patterns, functional validation can be challenging due to the high numbers of candidate variants. We designed an integrative approach combining a GWAS on *S. pneumoniae* clinical isolates, followed by whole-genome transformation coupled with NGS to functionally characterize a large set of GWAS candidates. Our study validated several phenotypic *folA* mutations beyond the standard Ile100Leu mutation, and showed that the overexpression of the *sulA* locus produces trimethoprim (TMP) resistance in *Streptococcus pneumoniae*. These validated loci, when used to build ML models, were found to be the best inputs for predicting TMP minimal inhibitory concentrations. Integrative approaches can bridge the genotype-phenotype gap by biological insights that can be incorporated in ML models for accurate prediction of drug susceptibility.

## INTRODUCTION

*Streptococcus pneumoniae* is a common Gram-positive commensal in the human upper respiratory tract but also an important pathogen that can cause infections ranging from mild diseases such as otitis and tonsillitis to life-threatening conditions such as pneumonia, sepsis, or meningitis. It represents a major cause of morbidity and mortality worldwide, especially in children, elders, and immunocompromised patients (1). Treatment and prevention of pneumococcal diseases is complicated by widespread antimicrobial resistance (AMR) and vaccine-escape events (2).

Trimethoprim (TMP), part of the WHO Model List of Essential Medicines (3), is a commonly used antibiotic. TMP is usually used in combination with sulfamethoxazole (SMX), and this combination is useful for treating a range of pneumococcal diseases (4). TMP inhibits the dihydrofolate reductase (DHFR) enzyme encoded by the gene *folA* (*dhfR*), while SMX inhibits the dihydropteroate synthase (DHPS) enzyme encoded by the gene *sulA* (*dhpS* or *folP*) (5). Both enzymes are necessary for the formation of tetrahydrofolate (THF), a cofactor to many metabolic reactions involved with amino acid and nucleic acid biosynthesis, and an important one-carbon donor (6, 7). While mostly used in combination, TMP alone is still used for urinary tract infections (3) and its use as a monotherapy was found as efficient as the combination in a number of infectious conditions (8, 9). This prompted us to decipher TMP resistance mechanisms in *S. pneumoniae*. We previously characterized mutants selected for TMP resistance *in vitro*, which allowed the discoveries of novel phenotypic *folA* mutations but also novel genes involved in resistance (10). We now extend our study of TMP resistance by investigating a large collection of resistant *S. pneumoniae* clinical isolates.

Mutations in *folA*, notably the FolA Ile100Leu substitution, are a key determinant of TMP resistance (11–16). Multiple additional mutations in *S. pneumoniae* FolA have been described but their exact role in the resistance phenotype warrants further investigations (13, 16, 17). Indeed, TMP resistance levels in *S. pneumoniae* frequently exceed those provided by the FolA Ile100Leu mutation (16–32 µg/mL) (10, 11) suggesting the presence of additional mutations contributing to TMP resistance.

The advent of next-generation sequencing (NGS) has allowed the development of innovative approaches to decode the genotype-phenotype relationships such as genome-wide gene function studies, genome-wide association studies (GWAS) and machine learning (ML). Genome-wide gene function studies applied to AMR mainly employed NGS in combination with chemogenomic screens such as chemical mutagenesis (Mut-seq) (10, 18, 19), transposon mutagenesis (Tn-seq) (20–22), step-wise selection on agar plates (Sel-seq) (23–25), or continuous selection in the morbidostat or similar devices (26–28) to identify genes or mutations enabling resistance. While genome-wide gene function studies rely on laboratory-generated mutants, GWAS explores associations between naturally occurring genetic variants and specific traits in populations. In *S. pneumoniae*, GWAS has been used to study AMR to different classes of antibiotics including beta-lactams, macrolides, fluoroquinolones, SMX, and TMP (29–32). However, results reported from GWAS are rarely supported by functional validation. ML-based AMR prediction algorithms often focus on assigning bacteria to binary phenotypes, that is, resistant or susceptible. This approach relies on breakpoints that can change over time and often does not capture the wide variation in the minimum inhibitory concentration (MIC) of antibiotics. An increasing number of ML models for MIC prediction are now emerging to overcome these limitations (33–41).

In this study, we designed an integrative approach to explore resistance to TMP in *S. pneumoniae*. First, we conducted GWAS on 662 *S*. *pneumoniae* genomes; second, we used resistance reconstruction by whole-genome transformation (WGT) coupled with NGS to functionally characterize GWAS candidates. WGT had the advantage of testing for a large number of candidates while offering the possibility of detecting large-scale genomic rearrangement. Shared recombination blocks highlighted by WGT were then specifically studied for their role in resistance by targeted transformation. We demonstrated that multiple additive mutations in the *folA* and *sulA* loci produce TMP resistance in *S. pneumoniae*. Finally, ML models were developed to predict TMP MIC in *S. pneumoniae*. The best digital MIC prediction models were based on the single nucleotide polymorphism (SNP) signatures of *folA* and *sulA* loci.

## RESULTS

### Population structure of the genome collection

A total of 662 *S*. *pneumoniae* isolates derived from three studies (25, 42, 43), for which whole genome sequencing and TMP MIC data were available, were used in this analysis. The TMP MIC values ranged from 0.064 to 2048 µg/mL, corresponding to a range of log_2_ values from −4 to 11. We used the EUCAST breakpoint MIC of 1 µg/mL as the cutoff value to define susceptibility to TMP (44), resulting in 417 TMP susceptible and 245 resistant strains (Fig. S1; Table S1A). Most resistant strains (170/245 isolates) were from Canada and China, while most of the susceptible ones (406/417 isolates) were from the United States (Fig. S1). The *in silico* predictions of sequence type (ST), serotype, and Global pneumococcal sequence cluster (GPSC) of the isolates are shown in Table S1A and summarized in Fig. S2. This data set contains 175 known STs and 24 new/undetermined STs (Table S1A). None of the STs encompass more than 10% of the strains with ST199 being the most frequent (*n* = 65; 9.8%) (Fig. S2A). Serotype analysis revealed 50 serotypes and 29 serogroups (Table S1A) with serogroup 19 (*n* = 116; 17.5%) and serotype 19A (*n* = 71; 10.7%) being the most frequent (Fig. S2B). Lineage analysis identified 75 GPSCs (Table S1A), with GPSC4 (*n* = 83; 12.5%) being the most prevalent (Fig. S2C). Different STs, serogroups, and GPSCs usually include resistant and sensitive isolates (Fig. S2).

### Genomic characterization of the genome collection

**Fig 1:**
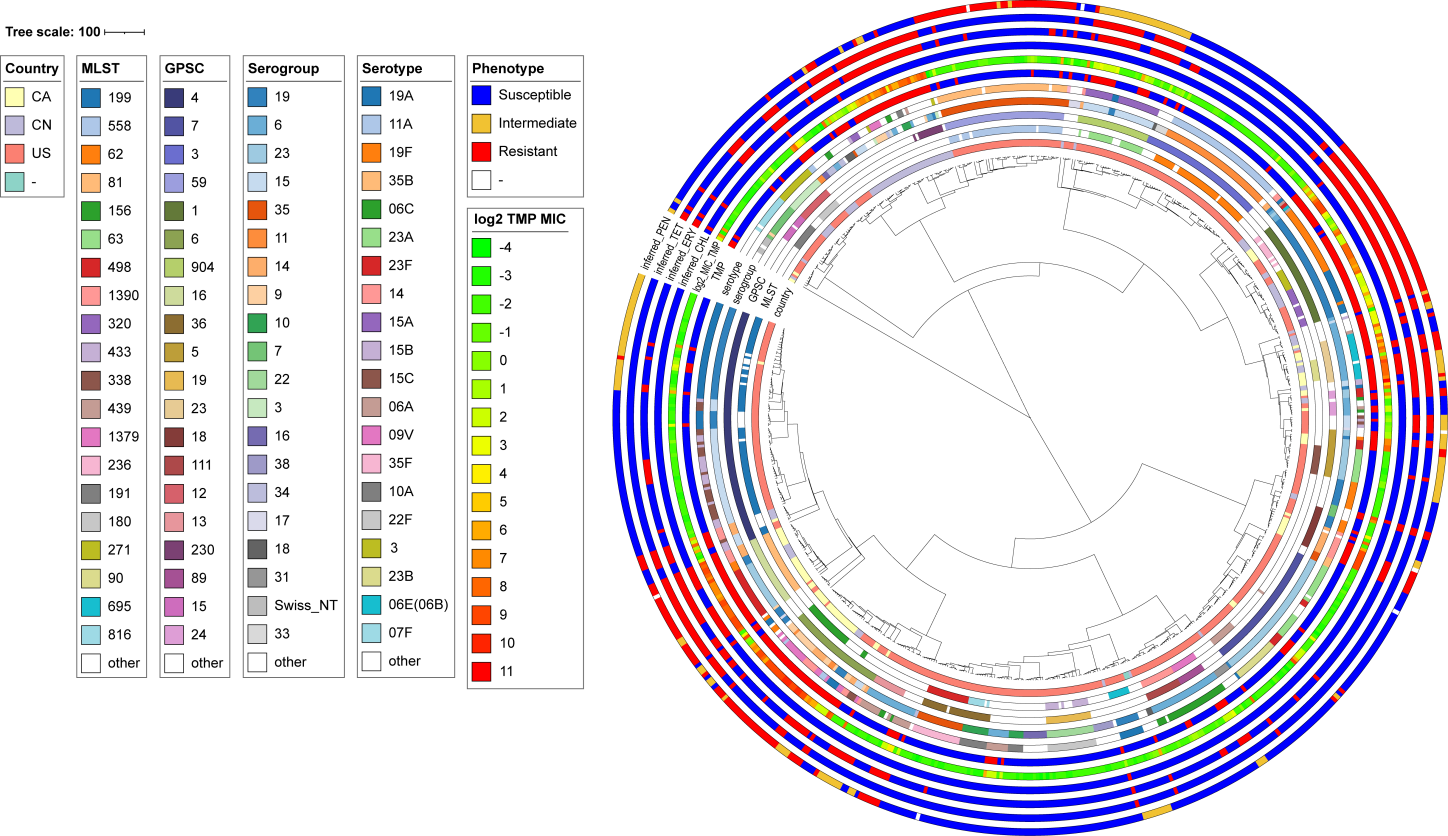
Recombination-free maximum-likelihood tree of the 662 *S*. *pneumoniae* strains used in this study. The tree was created using Gubbins (46), the tree scale bar represents the number of recombination-filtered substitutions across the genome. PEN, penicillin; TET, tetracycline; ERY, erythromycin; CHL, chloramphenicol; TMP, trimethoprim. This figure can be visualized interactively at https://microreact.org/project/h9uNDvre8DzWqgdZtUJeiZ-spntmp662march2024.

The *S. pneumoniae* genome assemblies ranged from 1.92 to 2.29 Mb (Table S1B), with 1,326,599 coding sequences (CDSs) annotated (Table S1C). The pangenome contained 5,166 clusters of orthologous genes (COGs), with 1,572 core genes (95% ≤ strains ≤ 100%), 587 shell genes (15% ≤ strains < 95%), and 3,007 cloud genes (0% ≤ strains < 15%) (Fig. S3). A total of 218,173 variant sites, including 187,258 SNPs in CDSs (115,876 synonymous and 71,382 non-synonymous) and 30,915 intergenic SNPs, were found compared to the reference *S. pneumoniae* D39V (45). A maximum-likelihood recombination-free phylogenetic tree constructed from the core genome SNPs alignment confirmed that isolates belonging to the same ST or GPSC were well clustered (Fig. 1). TMP resistance was highly correlated with inferred resistance to other antibiotics including chloramphenicol, erythromycin, tetracycline, and penicillin (Fig. 1; Table 1).

**TABLE 1:** Chi-squared test of association between TMP resistance and inferred resistance to other antibiotics among the 662 *S*. *pneumoniae* strains used in this study

Inferred resistance^*a*^	TMP resistance		Chi-squared statistic	*P* value
	Resistant	Susceptible		
Chloramphenicol			47.59	5.24 × 10^−12^
Resistant	32	2		
Susceptible	213	415		
Erythromycin			238.48	8.43 × 10^−54^
Resistant	182	59		
Susceptible	63	358		
Tetracycline			229.73	6.82 × 10^−52^
Resistant	157	35		
Susceptible	88	382		
Penicillin^*b*^			161.66	8.02 × 10^−35^
Resistant	129	45		
Intermediate	52	75		
Susceptible	62	292		
ND^*c*^	2	5		

^*a*^ Inferred resistance profiles were computed using the Pathogenwatch Antimicrobial Resistance Prediction module.

^*b*^ Penicillin susceptibility categories were based on oral penicillin CLSI breakpoints (47).

^*c*^ ND, not determined.

### Genome-wide association study to identify loci associated with TMP resistance

**Fig 2:**
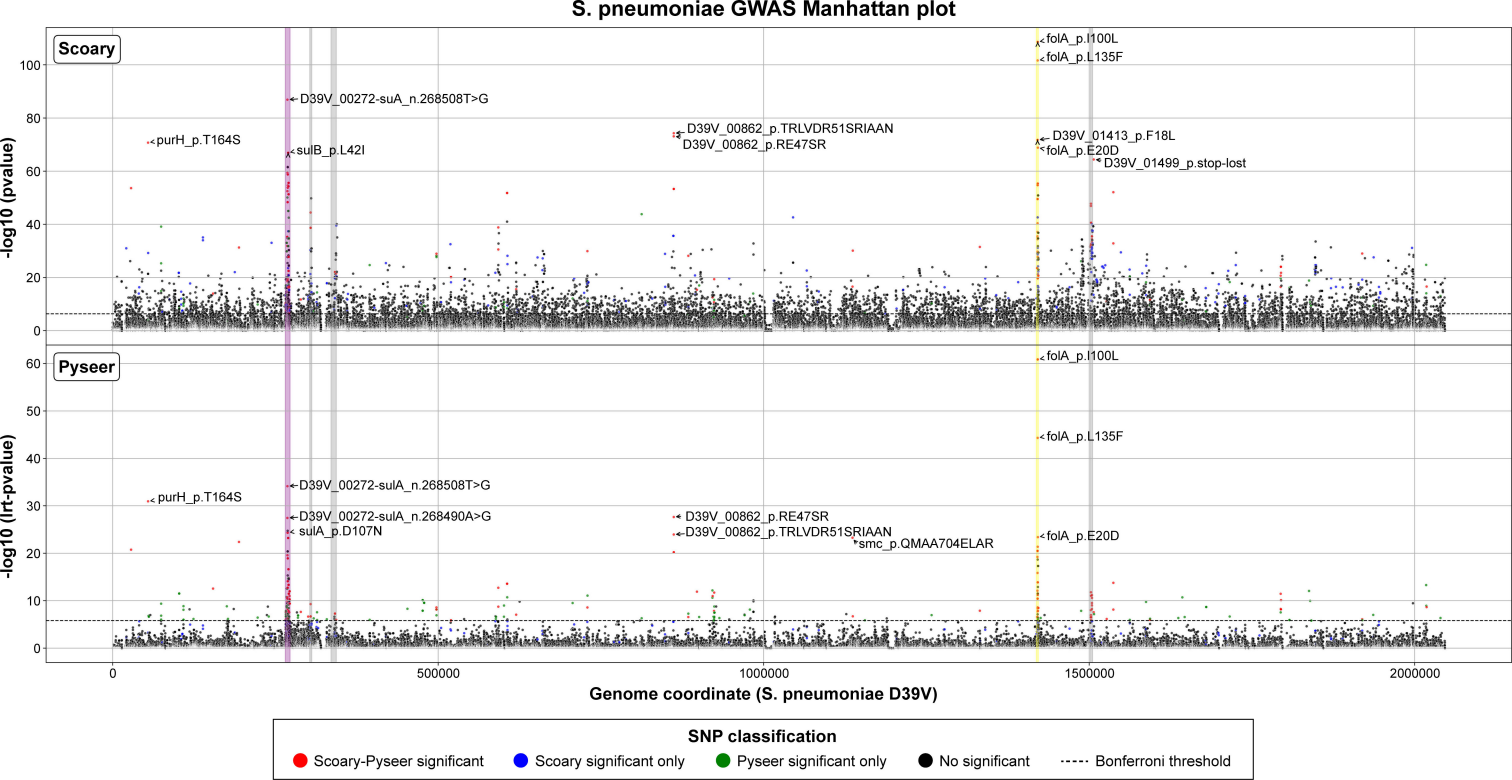
Manhattan plots summarizing the association of SNPs with the resistance to TMP according to Scoary (48) (top) and Pyseer (49) (bottom). Horizontal dotted lines indicate the Bonferroni-corrected threshold (α = 0.05). TMP resistance-associated SNPs detected by both approaches are colored in red, while significant SNPs detected by only Scoary or Pyseer are colored in blue and green, respectively. The *sulA* locus and the *folA* locus are highlighted in purple and yellow, respectively. The *pbp2x*, *pbp1a*, and *pbp2b* genes and their neighbors are highlighted in gray. The top 10 SNPs the most associated with TMP resistance are labeled. See Table S2B for more details.

GWAS was conducted with Scoary (48) and Pyseer (49) which, respectively, uses a binary phenotype (resistant/susceptible) and a continuous phenotype (i.e., log_2_ MIC values) to identify COGs or SNPs associated with TMP resistance. Scoary detected 14 COGs that were significantly associated with TMP resistance whereas Pyseer detected only one, with no overlap between the two software (Table S2A). Most of these COGs (12/15) were annotated as hypothetical proteins and 10 were part of a putative integrative and conjugative element (ICE) containing AMR genes unrelated to TMP resistance [*erm*, *cat*, and *tet*(*M*)] (Fig. S4). Scoary and Pyseer, respectively, revealed 330 and 227 SNPs associated with TMP resistance, of which 108 were common (Table S2B). Most SNPs were found in clusters of AMR loci: *sulA*, *folA*, or penicillin-binding proteins genes (*pbp2x*, *pbp1a*, and *pbp2b*) (Fig. 2; Table S2B). We defined the *sulA* locus as the region from D39V_00270 (encoding a hydrolase) to D39V_00276 (*sulD*) and the *folA* locus from D39V_01412 (*clpX*) to D39V_01415 (*dpr*); these two loci showing the strongest peaks as highlighted by K-mer association (Fig. S5). The FolA Ile100Leu substitution was the mutation most significantly associated with TMP resistance (Fig. 2). Besides these AMR loci, the Thr164Ser substitution in the purine biosynthesis protein PurH and two variants of the hypothetical protein D39V_00862 also had strong association with TMP resistance (Fig. 2; Table S2B). The complete list of SNPs associated with TMP resistance derived from Scoary and/or Pyseer can be found in Table S2B.

### Validation of TMP resistance-associated loci by whole genome transformation

We used WGT to reconstruct TMP resistance levels found in six *S*. *pneumoniae* clinical isolates, including one weakly (CCRI18414, MIC 8 µg/mL), two moderately (CCRI8881 and CCRI22088, MIC 32 µg/mL), and three highly (CCRI15681, CCRI15136, and CCRI22765, MIC 512–1,024 µg/mL) TMP-resistant strains. These strains covered 91 of the 108 candidate SNPs co-identified by Scoary and Pyseer (Table S2B). Genomic DNA (gDNA) from the six *S*. *pneumoniae* strains were independently transformed into the susceptible strain *S. pneumoniae* R6 and transformants were selected under varying concentrations of TMP.

**Fig 3:**
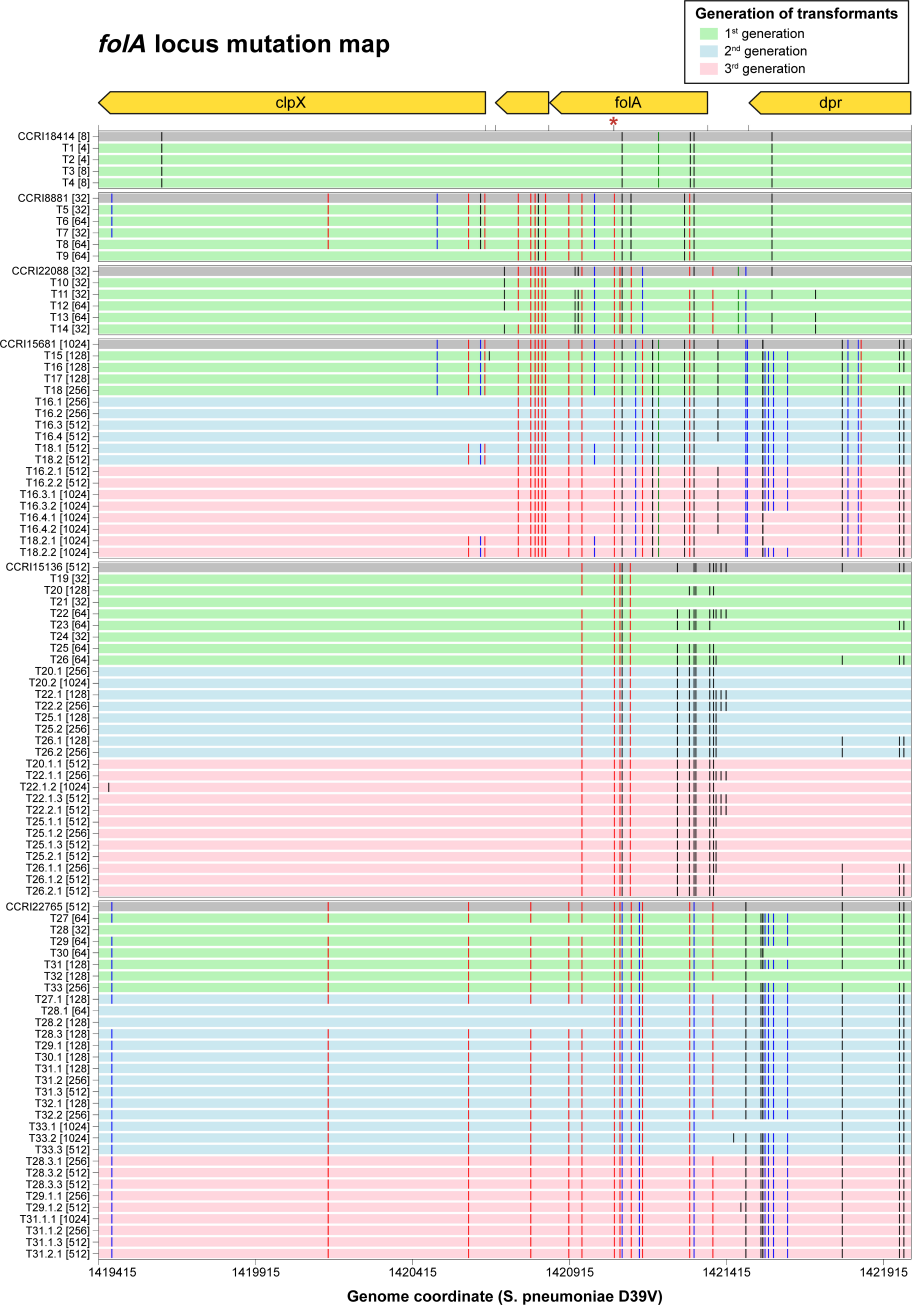
SNP map of the *folA* locus in the WGT-derived R6 transformants. SNPs are represented by vertical bars. SNPs detected as significant by Scoary and Pyseer are in red; SNPs detected as significant only by Scoary or Pyseer are in blue and green, respectively; not significant SNPs are in black. The FolA Ile100Leu mutation is marked by a red asterisk. TMP MICs are indicated within brackets next to the strains’ names. See Table S3 for the detailed list of SNPs per transformant.

**Fig 4:**
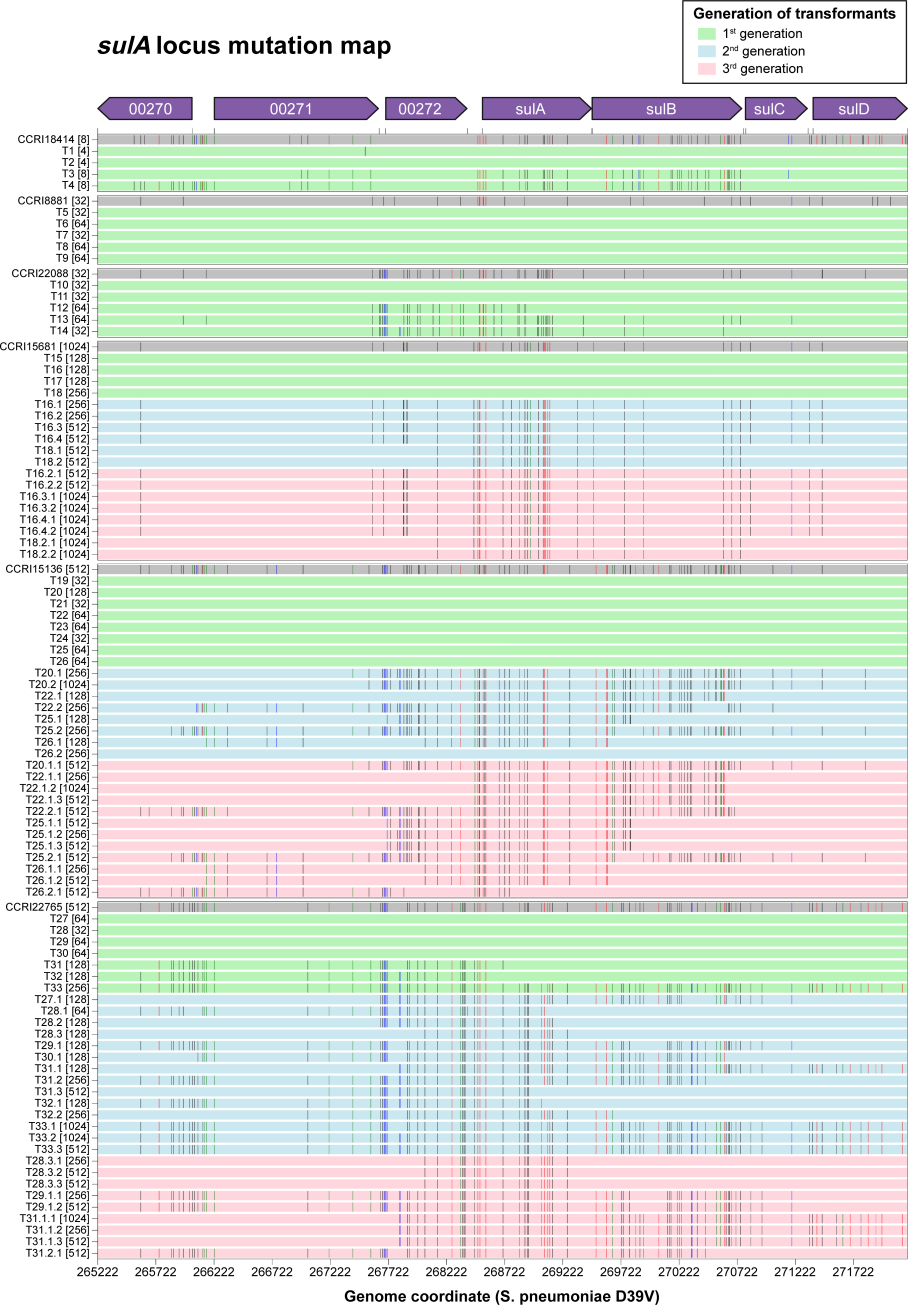
SNP map of the *sulA* locus in the WGT-derived R6 transformants. SNPs are represented by vertical bars. SNPs detected as significant by Scoary and Pyseer are in red; SNPs detected as significant only by Scoary or Pyseer are in blue and green, respectively; not significant SNPs are in black. TMP MICs are indicated within brackets next to the strains’ names. See Table S3 for the detailed list of SNPs per transformant.

A single round of WGT was sufficient to reconstruct the level of TMP resistance of strains with low or moderate resistance. Transformation of *S. pneumoniae* R6 with gDNA derived from CCRI18414 (MIC 8 µg/mL) led to transformants T1 to T4 (Fig. S6A). These had TMP MICs of either 4 µg/mL (T1 and T2) or 8 µg/mL (T3 and T4) (Fig. S6A). SNPs leading to the Leu16Val, Glu20Lys, Met53Ile, and Asp92Ala substitutions in FolA were transferred in the four transformants, although only Met53Ile was significant according to Pyseer (Fig. 3; Table S3A). SNPs in the coding regions of *sulA* and *sulB*, as well as in the intergenic region upstream of *sulA*, were detected only in T3 and T4, the two transformants with an MIC of 8 µg/mL (Fig. 4; Table S3A). *S. pneumoniae* transformants T5 to T9 were derived from CCRI8881 (MIC 32 µg/mL) (Fig. S6B) and acquired up to 11 SNPs in the *folA* locus reported as significant by both Scoary and Pyseer, including the FolA Ile100Leu mutation (Fig. 3; Table S3B). Transformants T5 and T7 had an MIC of 32 µg/mL, while T6, T8, and T9 had an MIC of 64 µg/mL (Fig. S6B), an MIC higher than the parent isolate. The three latter transformants had slightly more intergenic SNPs and T9 had more DNA transformation blocks (Table S3B). The transformants T10 to T14 were derived from CCRI22088 (MIC of 32 µg/mL) and acquired up to 12 significant SNPs co-detected by Scoary and Pyseer in the *folA* locus, including the FolA Ile100Leu mutation (Fig. S6C; Table S3C). Transformants T10, T11, and T14 had an MIC of 32 µg/mL, but T12 and T13 had an MIC of 64 µg/mL (Fig. S6C). This elevated TMP resistance in T12 and T13 could be explained by the transformation at the *sulA* locus of five SNPs co-detected by Scoary and Pyseer, although the same *sulA* locus mutations also transferred in T14 but with no increase in the MIC to TMP (Fig. 4; Table S3C).

Reconstruction of resistance for the more resistant isolates required up to three rounds of WGT but common patterns emerged. The first round of transformation with gDNAs derived from either CCRI15681, CCRI15136, or CCRI22765 led to transformants T15 to T33 which invariably acquired SNPs at the *folA* locus (Fig. 3; Table S3D through F). These transformants had a TMP MIC ranging from 32 to 128 µg/mL, although two (T18 and T33) had an MIC of 256 µg/mL. While all contained the FolA Ile100Leu mutation, in general higher TMP resistance came with more SNPs in *folA*, transformants with an MIC of 32 µg/mL having fewer SNPs than those with a MIC ≥64 µg/mL (Mann-Whitney test, *P* value 6.32 × 10^−3^, Fig. S7). Of note, the Leu135Phe mutation in FolA found in the three clinical isolates and co-detected by Scoary and Pyseer was transferred in all transformants except T21 and T28 (MIC 32 µg/mL) (Table S3D through F). For this first round of WGT, mutations at the *sulA* locus were transformed only in the most resistant transformants derived from CCRI22765 (T31 to T33, Fig. 4; Table S3F).

For the second round of transformation, a subset of transformants from the first round were transformed with relevant gDNAs. This led to transformants with an additional two- to fourfold resistance to TMP (Fig. S6D through F). Except for T26.2, the *sulA* locus was transferred in all these transformants as part of a DNA transformation block of 7–10 genes (*adhA* to *rpsI*, Table S3D through F; Fig. 4). Among the significant SNPs co-detected by Scoary and Pyseer in SulA, those leading to the mutations Val11Ala and Asp107Asn were transformed in all the second generation transformants except for the aforementioned T26.2, while Ala179Thr was only missing in T31.3, T32.1, T33.1, T33.2, and T33.3 (Table S3D through F). Transformation of significant TMP resistance-associated SNPs also occurred in the intergenic region upstream of *sulA* (Table S3D through F). The mutations in *sulA* known to be involved in sulfonamide resistance (one- or two-codon insertion within *sulA* [50]) were transferred in our transformants, albeit not being significantly associated with TMP resistance according to Pyseer and Scoary (Table S3D through F). Several significant SNPs co-detected by Scoary and Pyseer in *sulB* were also transferred to transformants derived from CCRI15136 and CRI22765 (Fig. 4; Table S3E through F).

A third round of transformation with the gDNAs of the highly resistant isolates led to transformants gaining an additional two- to fourfold resistance to TMP (Fig. S6D through F). In this round, no common new locus was transferred (Table S3D through F). However, we detected additional mutations in the *sulA* locus in several transformants (e.g., T22.2.1 or T26.2.1) (Fig. 4; Table S3E). We could not, however, pinpoint mutations potentially responsible for the increase in TMP resistance in most transformants. We assessed copy number variations (CNVs) using read count (RC) analysis with CNOGpro (51), which employs statistical methods including Hidden Markov Model and bootstrapping to detect regions where the normalized RCs deviate significantly from expected values. CNVs were identified as regions with significantly increased RCs (copy number gains or duplications) or significantly decreased RCs (copy number losses or deletions). Putative gene duplications detected in the transformants are summarized in Table S4. No CNV in *folA* or *sulA* was detected.

Overall, our WGT experiments showed that among the SNPs associated with TMP resistance (Table S2B), only SNPs in the *folA* and *sulA* loci were recurrently transformed into the transformants under TMP selection pressure, while the other SNPs were either not transformed or transformed only in a single WGT experiment (Table S3G). This prompted us to focus on the *folA* and *sulA* loci for further characterization.

### Targeted transformation

**Fig 5:**
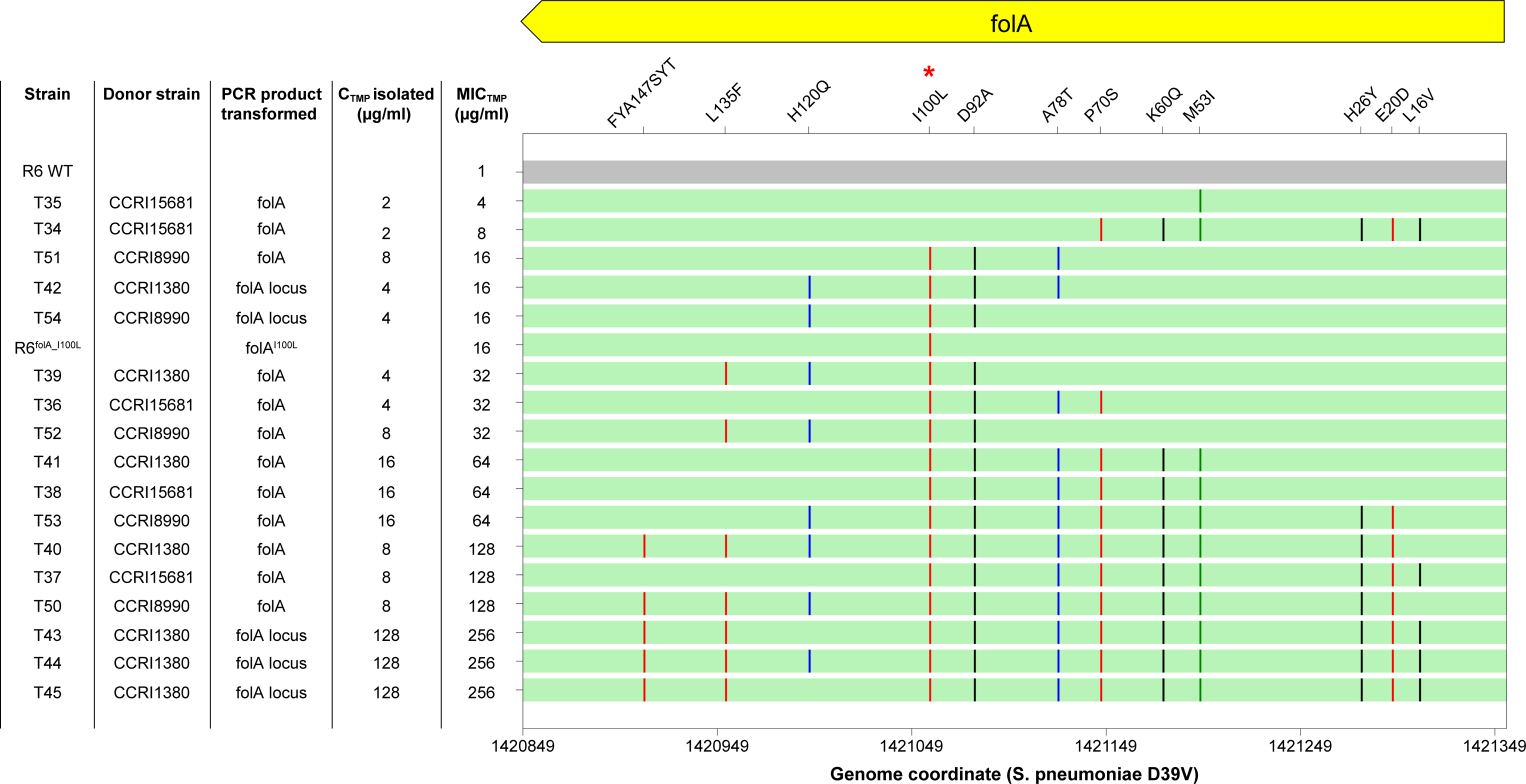
SNP map for the *folA* gene in the R6 transformants derived from targeted transformation. Each SNP is represented by a vertical bar: significant SNPs co-detected by Scoary and Pyseer are in red; significant SNPs detected by only Scoary or Pyseer are in blue and green, respectively; no significant SNPs are in black. The FolA Ile100Leu mutation is marked by a red asterisk.

To further validate the role of the *folA* and *sulA* loci in producing TMP resistance, we amplified either the *folA* locus (four genes) or the *folA* gene from gDNAs derived from CCRI15681 and from two other strains (CCRI1380 and CCRI8990) which had similar TMP MIC (1,024 µg/mL) and SNP profiles for the *folA* and *sulA* loci. The PCR products were transformed into *S. pneumoniae* R6 and transformants were selected on plates containing between 2 and 128 µg/mL of TMP (Table S5A). This led to transformants with MICs ranging from 4 to 256 µg/mL (Table S5A). The only transformants that grew on plates with 128 µg/mL of TMP were obtained from the transformation of the *folA* locus amplified from CCRI1380 (Table S5A). We sequenced the *folA* locus of three of these transformants with an MIC of 256 µg/mL (T43 to T45). While two transformants had SNPs in genes other than *folA* (*clpX* and *D39V_01413*), the third one (T43) acquired mutations exclusively in *folA* (Table S5B). We sequenced *folA* from a selection of our transformants with TMP MIC ranging from 4 to 128 µg/mL (Table S5A). One (T35) had an MIC of 4 µg/mL and the only SNP transferred was the FolA Met53Ile mutation (Fig. 5; Table S5B). This mutation was also found in the transformant with an MIC of 8 µg/mL (T34), along with mutations at amino acid positions 16, 20, 26, 60, and 70 (Fig. 5; Table S5B). All transformants with a TMP MIC ≥16 µg/mL had two common SNPs, Asp92Ala, and Ile100Leu. Transformants resistant to 16 µg/mL TMP, in addition to Asp92Ala and Ile100Leu, had acquired SNPs at either positions 78 or 120. The latter two mutations, as well as Asp92Ala, are unlikely to be phenotypic since transformation of a *folA* Ile100Leu PCR fragment (10) confers by itself an MIC of 16 µg/mL (Fig. 5). Transformants with an MIC of 32, 64, and 128 µg/mL had, respectively, 4, 6–9, and 9–11 mutations in *folA* (Fig. 5). Differences in FolA mutation profiles between transformants resistant to 32 and 16 µg/mL suggest that either Pro70Ser or Leu135Phe, along with Ile100Leu, could be phenotypic (Fig. 5; Table S5B). All transformants with a TMP MIC of 64 µg/mL shared three mutations (Ile100Leu, Pro70Ser, and Met53Ile) along with mutations at positions 60, 78, and 92 (Fig. 5; Table S5B), although the latter two are unlikely to be phenotypic as discussed above. Two of the three transformants with a TMP MIC of 128 µg/mL had SNPs leading to FolA Ile100Leu, Pro70Ser, Met53Ile, and Leu135Phe mutations (Fig. 5).

**Fig 6:**
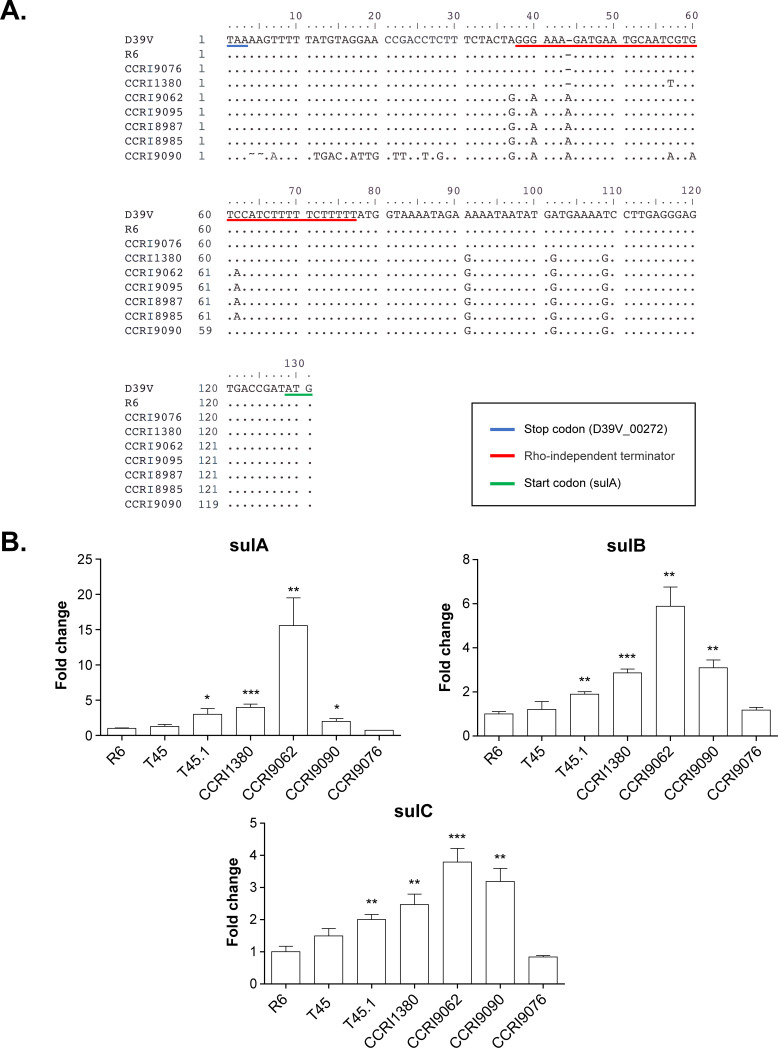
Impact of intergenic mutations upstream of *sulA* on the expression of the *sulABCD* operon. (**A**) Alignment of intergenic sequences between D39V_00272 (encoding a membrane-bound protease, CAAX family) and *sulA*. The stop codon of D39V_00272 and the start codon of *sulA* are underlined in blue and green, respectively. The putative Rho-independent terminator annotated by PneumoBrowse (45) (https://veeninglab.com/pneumobrowse) is underlined in red. (**B**) Gene expression levels of *sulA*, *sulB*, and *sulC* as determined by RT-qPCR. Data are shown as the mean fold change (with standard error) relative to *S. pneumoniae* R6 wild type (WT). All RT-qPCR data were normalized according to the amplification signals of the housekeeping gene *era* mRNA. ***P < 0.001, **P < 0.01, *P < 0.05 as determined by *t* test.

We next investigated the contribution of the *sulA* locus in TMP resistance. No transformants could be obtained when using the PCR products of the genes *sulA*, *sulB*, *sulC*, or *sulD* individually or by co-transformation, in contrast to the transformation of the *sulA* locus which increased the TMP MIC of *S. pneumoniae* R6 by twofold (see T46-T49 in Table S5A and C). We also transformed the *sulA* locus in the three transformants in which we had previously transformed the *folA* locus derived from CCRI1380 and resistant to 256 µg/mL TMP (T43 to T45). This led to transformants resistant to 512 µg/mL TMP (see T43.1, T43.2, T44.1, T44.2, T45.1, and T45.2 in Table S5A and C). We sequenced the *sulA* locus for these ten transformants; eight had between 27 and 35 SNPs in multiple genes but two (T45.1 and T45.2) had only nine SNPs: one in a protease of the CAAX family, four in the intergenic region between this protease and *sulA,* and four impacting amino acids up to Asp107Asn in SulA (Table S5C). Intriguingly, the transformant T43.2 had the four mutations upstream of *sulA* but lacked those SNPs in the N-terminal region of SulA (Table S5C). This intergenic region revealed a predicted Rho-independent terminator (Fig. 6A) and we postulated that intergenic SNPs upstream of *sulA* might disturb its structure and impact the expression of the *sulABCD* operon. To test this, we carried out reverse transcription-quantitative PCR (RT-qPCR) for the first three genes of the *sul* operon in a transformant that harbored the four SNPs upstream of *sulA* in addition to SNPs in *folA* (T45.1), its parent strain with SNPs only in *folA* (T45) and the clinical strain CCRI1380. As a negative control, we included the clinical strain CCRI9076 with no intergenic SNPs upstream of *sulA*. As expected, we observed increased expression of *sulA*, *sulB*, and *sulC* in T45.1 and CCRI1380 but not in T45 or CCRI9076 (Fig. 6B).

### Recombination analysis shed light on the origin of TMP resistance in *S. pneumoniae*

We used Gubbins (46) to identify genomic regions containing elevated densities of SNPs, an indication of homologous recombination. We concentrated the analysis on the *sulA* and *folA* loci. Recombination blocks spanning the *sulA* locus were detected in all strains of this study (Fig. S8). Similarly, recombination blocks were detected in the *folA* locus of most of the TMP-resistant strains (240/245 isolates, Fig. S9). We found, however, in five resistant clinical strains an absence of any recombination block in the *folA* locus (Fig. S10). These five strains (CCRI9062, CCRI9095, CCRI8987, CCRI8985, and CCRI9090), with a TMP MIC of 128–256 µg/mL, only contain three SNPs in FolA compared to D39V (Ile100Leu, Leu16Val, and Asp92Ala). While the Ile100Leu mutation produces TMP resistance to 16 µg/mL (Fig. 5), the latter two are unlikely to be phenotypic as they were ubiquitous in *S. pneumoniae* clinical isolates: Leu16Val was present in 206/245 resistant and 414/417 susceptible strains and Asp92Ala was present in 146/245 resistant and 245/417 susceptible strains. These five strains contain three to four mutations within the putative Rho-independent terminator upstream of *sulA*, with four strains sharing the same SNP profile (Fig. 6A). We thus conducted RT-qPCR of *sulA*, *sulB*, and *sulC* in CCRI9062 and CCRI9090. Consistent with our work with targeted transformation, we observed an increased expression for these genes in these two strains that harbor SNPs upstream of *sulA* (Fig. 6B).

### Machine learning to predict TMP MIC

We built ML models to predict TMP MIC from SNPs by both regression and multi-class classification approaches, evaluating common ML algorithms with default parameters. In total, 31 models were tested, including 16 regression and 15 classification models. When applied to our data set (662 genomes), all classification models except Decision Tree performed better than their corresponding regression models (Fig. S11). All models, except four regression models, performed better than the baseline (dummy model), and the highest within one-tier accuracy rate (85.5%) was achieved by the CatBoost, LightGBM, and XGBoost classification models (Table 2; Table S6A and B; Fig. S12). We also built models that used only SNPs in either *folA* or the *folA* and *sulA* loci as input. In general, the models using both loci performed better than those using only *folA*; they also performed equally or better than those using the whole-genome SNPs (Table S6A and B; Fig. S13). Among all built models, the logistic regression and linear support vector classification models using SNPs from the *folA* and *sulA* loci gave the highest within one-tier accuracy rate (86.3%) (Table 2; Fig. S14). We also evaluated the models based on the major error rate (MER), defined as the rate of susceptible isolates having incorrectly predicted resistant MICs, and the very major error rate (VMER), defined as the rate of resistant isolates having incorrectly predicted susceptible MICs. The FDA standards for automated systems require (i) a within one-tier accuracy rate >89.9%, (ii) an MER ≤ 3%, and (iii) a lower and upper 95% confidence limit for the VMER of ≤1.5% and ≤7.5%, respectively (52). Our two best models had an MER ≤3% and an upper 95% confidence limit for the VMER ≤5%, which align with FDA standards (Table 2). However, their within one-tier accuracy rate (86.3%), as well as their lower 95% confidence limit for the VMER (3.9% and 4.0%) did not meet the FDA criteria (Table 2). Attempts to optimize these models by hyperparameter tuning (Table S6C) and feature selection methods using chi-squared test, ANOVA *F*-value, and recursive feature elimination (53) failed to improve the models, however.

**TABLE 2:** Performance of the best machine learning models for TMP MIC prediction according to the input used

Input	Model^*a*^	One-tier accuracy^*b*^		MER^*c*^		VMER^*d*^	
		Average	95% CI^*e*^	Average	95% CI^*e*^	Average	95% CI^*e*^
All SNPs	CatBoost	0.855	(0.849–0.862)	0.028	(0.024–0.031)	0.043	(0.038–0.047)
	LightGBM	0.855	(0.848–0.861)	0.028	(0.025–0.032)	0.046	(0.042–0.050)
	XGBoost	0.855	(0.848–0.862)	0.030	(0.027–0.034)	0.042	(0.038–0.046)
*folA* SNPs	Extra-trees	0.810	(0.803–0.817)	0.012	(0.010–0.015)	0.060	(0.056–0.064)
*folA* and *sulA* loci SNPs	Logistic regression	0.863	(0.857–0.870)	0.027	(0.024–0.030)	0.045	(0.040–0.050)
	Linear support vector	0.863	(0.856–0.869)	0.030	(0.027–0.033)	0.043	(0.039–0.047)

^*a*^ All models shown in this table are classification models.

^*b*^ Accuracy within ±1 twofold dilution factor, based on a 10 times-repeated stratified fivefold cross-validation.

^*c*^ Major error rate, defined as susceptible genomes predicted to be resistant.

^*d*^ Very major error rate, defined as resistant genomes predicted to be susceptible.

^*e*^ 95% CI, 95% confidence interval.

## DISCUSSION

We built an *S. pneumoniae* genome collection associated with TMP MIC data where we integrated GWAS, resistance reconstruction by transformation coupled with sequencing, and ML approaches for an in-depth investigation of TMP resistance in *S. pneumoniae*. Microbial GWAS applied to AMR studies first treated phenotypes as a binary trait—resistant or susceptible, similar to the case-control design in human GWAS (29, 54–58). More recently, microbial GWAS based on the quantitative trait of MIC was used to identify variants that cause more subtle changes in antibiotic susceptibility (32, 59, 60). Two GWAS studies using the binary trait-based method dealt with TMP resistance in *S. pneumoniae* (30, 31). Population structure is one of the main sources of confounders in GWAS (61). To control for population structure, a range of tools have been developed with different analytical approaches, however no gold standard solution has been established (62, 63). In this study, our sample collection for GWAS was biased toward TMP-susceptible isolates, which were mainly from the United States (Fig. S1). While the strains isolated from the same country were not clustered together in the phylogenetic tree (Fig. 1), this country-biased sampling may still introduce confounding variables such as differences in antimicrobial usage practices and host or environmental factors. TMP resistance was indeed highly correlated with inferred resistance to other antibiotics in our data set (Fig. 1; Table 1). Yet comparing our GWAS results with those from other studies could help to enhance the reliability of the detected associations, as consistent findings across independent data sets suggest that the associations are less likely to be spurious. Here, we used both binary trait-based (with Scoary) and quantitative trait-based (with Pyseer) GWAS to gain insight into TMP resistance in *S. pneumoniae*. The two methods generated a list of 108 common SNPs associated with TMP resistance, which were mainly clustered in the *folA*, *sulA*, and *pbp* loci (Fig. 2; Table S2B). This is congruent with previous binary trait-based TMP GWAS studies in S. *pneumoniae* (30, 31). Our experimental reconstruction, using WGT experiments, showed that only the *folA* and *sulA* loci were recurrently transferred into the transformants, while *pbp* genes could be excluded and are likely to be hitchhikers i.e. associated but not causative SNPs. This is also probably the case for other SNPs strongly associated with TMP resistance such as the *purH* Thr164Ser mutation (Fig. 2; Table S2B). Different mechanisms could be involved for this hitchhiking. In case of *pbp* genes, association with TMP resistance suggests a strong co-selection for resistance to TMP and beta-lactams, probably due to antibiotics selection in the population, resulting in the emergence of multi-drug resistant (MDR) isolates (29, 30, 64). Hitchhiking mutations could be due to genetic linkage (65) or arise as a result of hypermutation, a phenomenon often associated with the emergence of AMR (66–68). Another possibility is that some mutations may provide a fitness advantage or act as compensatory mutations (69–71). A full exploration of the hitchhiking SNPs would give new insights into the genetic evolution and epistatic interaction of resistance in *S. pneumoniae*.

The FolA Ile100Leu substitution was confirmed to be the key mutation involved in TMP resistance by both our binary trait-based and our MIC-based GWAS (Table S2B). Recombination analysis allowed us to determine that this phenotypic SNP has evolved by recombination, as suggested by Adrian et al. (11), for most isolates (Fig. S9), or more rarely by spontaneous mutations, as suggested by Pikis et al. (12) (Fig. S10). Our GWAS analysis coupled with functional testing confirmed, or excluded, the role of additional mutations in FolA in TMP resistance. For example, the FolA mutation Asp92Ala highlighted in some studies (e.g., Cornick et al. [16])] was ruled out by our GWAS analyses (Table S2B); it was indeed ubiquitous in *S. pneumoniae* clinical isolates (present in 146/245 resistant and 245/417 susceptible strains). Interestingly, this mutation was always co-transformed along with the mutation Ile100Leu in all our transformation experiments, a likely case of hitchhiker in linkage disequilibrium with the causal SNP. The candidate FolA mutation Met53Ile, reported by Maskell et al. (13) that we validated *in vitro* (10), was retained by the MIC-based but not the binary trait-based GWAS (Table S2B). This mutation was transferred in both the WGTs of the low-resistant strain CCRI18414 and the highly resistant strain CCRI15681 (Fig. 3; Table S3A and D) as well as by our targeted transformation of *folA* in transformants that exhibited a 4 (or 8)-fold increase in their MICs to TMP (Fig. 5). Transformants that contained both the mutations Met53Ile and Ile100Leu in FolA showed higher TMP MIC than those that contained only one of these two mutations (Fig. 5). Similarly, our WGTs and our targeted *folA* transformation highlighted the role of FolA Leu135Phe and Pro70Ser mutations in contributing to TMP resistance (Fig. 5). Importantly, these two latter SNPs were found to be significantly associated with TMP resistance by both Scoary and Pyseer (Table S2B). In some WGT reconstruction experiments, the TMP MIC was higher in the last step transformants than in the initial parental resistant strains (e.g., Fig. S6B and C). This may have to do with the different genetic backgrounds between the receiving strain (R6) and the clinical isolates where the strength of the phenotype may be context-dependent. Future experiments that deal with more recipient and/or donor strains may help to better characterize the effect of each SNP and their combinations in different genomic contexts.

Our GWAS and those of others (30, 31) highlighted a cluster of TMP resistance-associated SNPs in the *sulA* locus (Fig. 2; Table S2B). Since TMP and SMX are often used in combination, it was suggested to be a result of a co-selection for resistance to these two drugs (31). In *S. pneumoniae*, mutations in *sulA* are well known to produce SMX resistance (50, 72, 73). However, it has been shown in *Staphylococcus aureus* that some mutations in *sulA* (DHPS) while leading to SMX resistance also increased TMP susceptibility (70). Furthermore, the cyclic mutual potentiation effects between TMP and SMX suggest that TMP and SMX susceptibility could be modulated by the metabolic flux and regulation of the folate biosynthesis pathway (74). SulABCD is upstream of FolA in the tetrahydrofolate biosynthesis pathway (Fig. S15). Our WGT experiments of different *S. pneumoniae* clinical strains showed that transformants harboring both the *sulA* and *folA* loci had higher TMP MIC than those harboring only the *folA* locus (Fig. 3 and 4; Table S3). Our targeted transformation experiment further validated that the introduction of the *sulA* locus conferred a twofold increase in TMP MIC (Table S5A and C). The mutations responsible for resistance are found in an intergenic region upstream of the *sulABCD* operon and lead to its increased expression (Fig. 6). This is in line with the results of an overexpression library in *S. pneumoniae* that showed that the simultaneous overexpression of *sulB* and *sulC* conferred a twofold increase in TMP MIC (10). As suggested (10), an increase in the metabolic flux in the folate biosynthesis pathway could modulate the susceptibility to TMP. These results also highlight the role of regulatory sequences modulating gene expression as a driver of antibiotic resistance in clinical isolates.

While our GWAS analyses combined with *in vitro* work have shown their potential to separate causal from hitchhiking SNPs and to determine the effect of each SNP and their multiple combinations on TMP MIC, machine learning could be a fast and efficient alternative for MIC prediction. Machine learning has been applied to predict the MICs of several antibiotics in *S. pneumoniae* (33, 34, 75, 76) but, at least for TMP, this is based on a binary classification that assigns an isolate as resistant or susceptible based on the presence/absence of the key FolA Ile100Leu mutation (https://github.com/pathogenwatch/amr-libraries). We thus carried out an exploratory study by evaluating the performance of different ML models using different inputs to predict not only whether strains are sensitive or resistant to TMP but also their TMP MICs (Table 2; Table S6A and B). Our results showed that prediction performance differed considerably between algorithms and input features. The best prediction performance was achieved by the linear support vector or logistic regression classification model using SNPs from the *folA* and *sulA* loci as input. Interestingly, these two models performed significantly better when using only these SNPs than when using the whole-genome SNPs as input (Fig. S13A). Compared to a typical machine learning study, our approach stands out by employing a biological rather than computational method for feature selection. Indeed, the choice of the *folA* and *sulA* loci SNPs as input was guided by our GWAS analyses and experimental validation. This suggests that biological guidance could pay off in building better prediction models. Contemporary data sets, once available, would be useful to evaluate the flexibility of the models. Improvements are needed before it could be used as a diagnostic tool (52). Indeed, our data set was imbalanced with nearly two-thirds of the strains being TMP susceptible. We could expect VMER to decrease when more resistant genomes are available (37). On the other hand, not all of the GWAS candidates have been investigated by our experimental validation (Table S2B). For example, significant TMP resistance-associated SNPs were identified in several transformants (e.g., SNPs in the acetyltransferase D39V_00191 or in the *recU* gene D39V_00342 in CCRI15681, or in the ABC transporter gene D39V_01811 in CCRI22765; see Table S3G); however, their exact contribution to TMP resistance awaits further experimental validation. Of note, our resistance reconstruction experiments sometimes failed to fully explain the TMP resistance level. While we focused on recurrent patterns, it is possible that mutations involved in resistance could be strain or transformant-specific. Furthermore, as the susceptible strain used in our resistance reconstruction experiments (*S. pneumonia* R6, TMP MIC 1 µg/mL) has a MIC higher than most susceptible clinical isolates (Fig. S1), our findings may overlook additional genetic determinants contributing to TMP resistance, particularly those specific to low-MIC strains. Future work may help in detecting additional SNPs involved in TMP resistance and these, once included in ML models, may lead to more accurate MIC assessment.

In summary, we provided an in-depth view of resistance to TMP in *S. pneumoniae* that extends the model based only on the FolA Ile100Leu mutation. We validated the role of several *folA* mutations and discovered the overexpression of the *sulA* locus as a genetic determinant of TMP resistance in *S. pneumoniae*. A model of TMP MIC prediction in *S. pneumoniae* based on SNP signatures in these two causal loci was created. Our roadmap from *in silico* analysis through experimental validation to diagnostic tool building could be adapted, where applicable, to explore AMR in different microorganisms and/or different drugs. Indeed, the decreasing cost of sequencing, along with the concomitant increase of publicly available genomes set the stage for GWAS (core SNPs-based or pangenome-based). Functional validation of GWAS candidates would provide insight into the molecular mechanisms underlying AMR while also helping to build accurate and interpretable prediction models.

## MATERIALS AND METHODS

### Culture conditions and MIC determination

*S. pneumoniae* strains were grown at 35°C with 5% CO_2_ in brain heart infusion (BHI; Becton Dickinson) or C+Y (77) broth, or on Trypticase soy agar supplemented with 5% sheep blood (TSAII, Becton Dickinson). MICs of TMP (Sigma) were determined by microdilution in 96-well plates in 0.1 mL cation-adjusted Müller-Hinton broth (Becton Dickinson) with 5% lysed sheep blood from at least three independent biological replicates.

### Genome sequencing

Genomic DNAs (gDNAs) were extracted from mid-log phase cultures using the Wizard Genomic DNA Purification Kit (Promega). Illumina Nextera XT sequencing libraries were prepared from gDNAs according to the manufacturer’s instructions. The size distribution of the libraries was validated using a 2100 Bioanalyzer and high-sensitivity DNA chips (Agilent Technologies). Sequencing was performed on an Illumina MiSeq, HiSeq2500, or NovaSeq6000 platform.

### DNA transformation

*S. pneumoniae* transformation was performed as previously described (78). gDNAs or PCR products (PCR primers listed in Table S7) from TMP-resistant *S. pneumoniae* were transformed into *S. pneumoniae* R6 and selected on a series of casein tryptone (CAT) agar plates supplemented with 5% (vol/vol) sheep blood and TMP at concentrations ranging from 2 to 1,024 µg/mL. PCRs were performed using the Phusion enzyme (Thermo Scientific). MICs were determined for transformants growing on higher TMP concentrations than mock-transformed controls. For PCR product transformation, mutations in transformants were validated by Sanger sequencing. For WGT, the genome of transformants was sequenced using the Illumina NovaSeq6000 platform.

### RNA extraction and RT-qPCR

RNA extraction and RT-qPCR were performed as described previously (79). All RT-qPCR data were normalized according to the amplification signals of the housekeeping gene *era*. The RT-qPCR primers are listed in Table S7.

### Genome collection

Eighty *S. pneumoniae* clinical isolates from the Collection du Centre de Recherche en Infectiologie (CCRI; Quebec City, QC, Canada) previously sequenced in our laboratory (25) were used in this study. In addition, we included 481 *S*. *pneumoniae* genomes from a collection of pneumococcal clinical isolates from Massachusetts, USA (42) and 99 *S*. *pneumoniae* genomes from the China National Microbiology Data Center (NMDC) (43) for which TMP MIC data were available. We also included the genomes of *S. pneumoniae* R6 (80) (GenBank accession number AE007317) and *S. pneumoniae* D39V (45) (GenBank accession number CP027540) (Table S1A).

### Genomic analyses

Genomes were assembled *de novo* using Spades (v3.13.0) with default parameters (81). The assemblies were then filtered to remove short (<1,000 bp) contigs. Assembly metrics were calculated using QUAST (v5.0.2) (82) and genome quality was assessed through checkM (v1.1.3) (83) using the lineage_wf workflow. Final genome assemblies were annotated using Prokka (v1.14.6) (84) with default parameters. Pangenome analysis was performed using Roary (v3.13.0) (85) with a minimum blastp identity of 90% and a threshold of 95% isolates for annotating a gene as a core gene. SNPs were detected from fastq sequencing reads using Snippy (v4.6.0) (https://github.com/tseemann/snippy) which wraps bwa-mem (86) for read mapping and freebayes (87) for variant calling (parameters: --minqual 100 --mincov 10 --minfrac 0.9). The genome of *S. pneumoniae* D39V (45) was used as reference, its annotation by Prokka is showed in Table S1D. Gubbins (v2.4.1) (46) was used to detect recombinant regions and generate phylogenetic tree (parameters: --tree_builder raxml --raxml_model GTRCAT --iterations 10). Sequence typing, serotyping, and GPSC assignment were performed using PubMLST (88), SeroBA (89), and PopPUNK (90), respectively, which were implemented in the Pathogenwatch platform (https://pathogen.watch). Inferred resistance profiles were computed using the Pathogenwatch Antimicrobial Resistance Prediction module (https://cgps.gitbook.io/pathogenwatch/technical-descriptions/antimicrobial-resistance-prediction). CNV analysis was performed using CNOGpro (51) and ICE detection was performed using ICEberg (91).

### Genome-wide association study

Genome-wide association study was conducted using Scoary (v1.6.16) (48) and Pyseer (v1.3.9) (49). COG and SNP presence/absence matrices were used as variant input. In addition, a k-mer based GWAS was performed using Pyseer. K-mers with lengths between 9 and 100 bases and with allele frequencies between 5% and 95% were extracted using fsm-lite (v1.0) (92). In Scoary, the TMP susceptibility phenotype (resistant or susceptible) was used as the outcome variable. Isolates having log_2_ MIC ≥1 were considered resistant. Associations in Scoary were scored using Fisher’s exact test, multiple testing corrections were carried out using the Bonferroni (93) and Benjamini-Hochberg (94) methods, population structure correction was conducted based on the phylogenetic tree using the pairwise comparison algorithm (95, 96), and a *post hoc* label-switching permutation test was run with 1,000 permutations. A variant was classified as associated with TMP resistance if (i) the Bonferroni corrected *P* value was <0.05, (ii) the worst pairwise comparison *P* value was <0.05, (iii) the empirical *P* value based on 1,000 permutations was <0.05, and (iv) the odds ratio >1. In Pyseer analysis, the log_2_ value of TMP MICs was used as the outcome variable. Associations in Pyseer were investigated using the linear mixed model (LMM) (97) to account for population structure and using a Bonferroni correction (α = 0.05) with the number of unique variant patterns as the number of multiple tests. The distance matrix for LMM was extracted from the phylogenetic tree using the Pyseer’s script phylogeny_distance.py. Pyseer outputs a beta value, that is, the effect size (or the slope of the regression line) for each variant with its associated likelihood ratio test (lrt) *P* value. We only considered variants positively associated with TMP resistance, that is, beta value >0. Pyseer’s lrt *P* value thresholds for significance post-Bonferroni correction were 3.77 × 10^−5^ for COGs, 1.61 × 10^−6^ for SNPs, and 2.51 × 10^−8^ for k-mers.

### Machine learning

#### Data preparation

MIC values were cleaned to remove the >, <, ≥, and ≤ symbols according to the following rules: (i) if the MIC was >*x*, the MIC was changed to 2*x*; (ii) if it was <*x*, the MIC was changed to x/2; and (iii) if the MIC was ≥*x* or ≤*x*, the symbol was removed and the MIC remained unchanged. The log_2_ values of TMP MICs, rounded to the nearest integer, were used as labels for all ML models.

#### Model generation

The prediction of MICs can be solved as a regression or a multi-class classification problem. SNP presence/absence matrices were used as input features; synonymous SNPs and singleton SNPs were filtered out. We investigated several regression and/or classification algorithms (98–110) (see Table S6A and B) from the Scikit-learn (v1.0.2) (111), the CatBoost (v1.0.6), the LightGBM (v3.2.1), and the XGBoost (v1.0.2) libraries. Dummy (baseline) model used the “most_frequent” strategy (i.e., always predicts the most frequent class in the training set) in case of classification and “median” strategy (i.e., always predicts the median of the training set) in case of regression.

#### Model evaluation

Model performance was evaluated using a 10 times-repeated stratified fivefold cross-validation (Fig. S16). The raw accuracy, the accuracy within ±1 twofold dilution factor (or one-tier), the major error rate (MER, i.e., susceptible isolate having incorrectly predicted resistant MIC), the very major error rate (VMER, i.e., resistant isolate having incorrectly predicted susceptible MIC) were computed along with the 95% confidence intervals for each model.

#### Model optimization

Hyperparameter tuning was performed using grid search or randomized search (112). Feature selection was performed using chi-squared test, ANOVA *F*-value, or recursive feature elimination (RFE) (53). While performing model optimization, model performance was evaluated using a nested cross-validation scheme (Fig. S17).

## Data Availability

The NGS data have been deposited in the Sequence Read Archive (SRA) database under the BioProject accession PRJNA1050271, sample accessions SAMN38729981 to SAMN38730070 (Table S8).

## SUPPLEMENTAL MATERIAL

Supplemental Figures mbio.01360-24-s0001.pdf Figures S1 to S17.

Table S1 mbio.01360-24-s0002.xlsx *S. pneumoniae* isolates.

Table S2 mbio.01360-24-s0003.xlsx COGs and SNPs associated with TMP resistance.

Table S3 mbio.01360-24-s0004.xlsx SNPs transformed in the WGT experiments.

Table S4 mbio.01360-24-s0005.xlsx Putative gene duplications detected by CNOGpro.

Table S5 mbio.01360-24-s0006.xlsx TMP MICs of and SNPs transformed in the R6 transformants.

Table S6 mbio.01360-24-s0007.xlsx Performance of classification and regression models for TMP MIC prediction.

Table S7 mbio.01360-24-s0008.xlsx Primers.

Table S8 mbio.01360-24-s0009.xlsx SRA BioSample accession numbers.

ASM does not own the copyrights to Supplemental Material that may be linked to, or accessed through, an article. The authors have granted ASM a non-exclusive, world-wide license to publish the Supplemental Material files. Please contact the corresponding author directly for reuse.
