# Supplementary material for: Holistic understanding of trimethoprim resistance in Streptococcus pneumoniae using an integrative approach of genome-wide association study, resistance reconstruction, and machine learning
Source: mBio. 2024 Aug 9;15(9):e01360-24. doi: 10.1128/mbio.01360-24 (PMC11389379; doi:10.1128/mbio.01360-24)
Supplement: Supplemental Figures — Figures S1 to S17. [file mbio.01360-24-s0001.pdf]

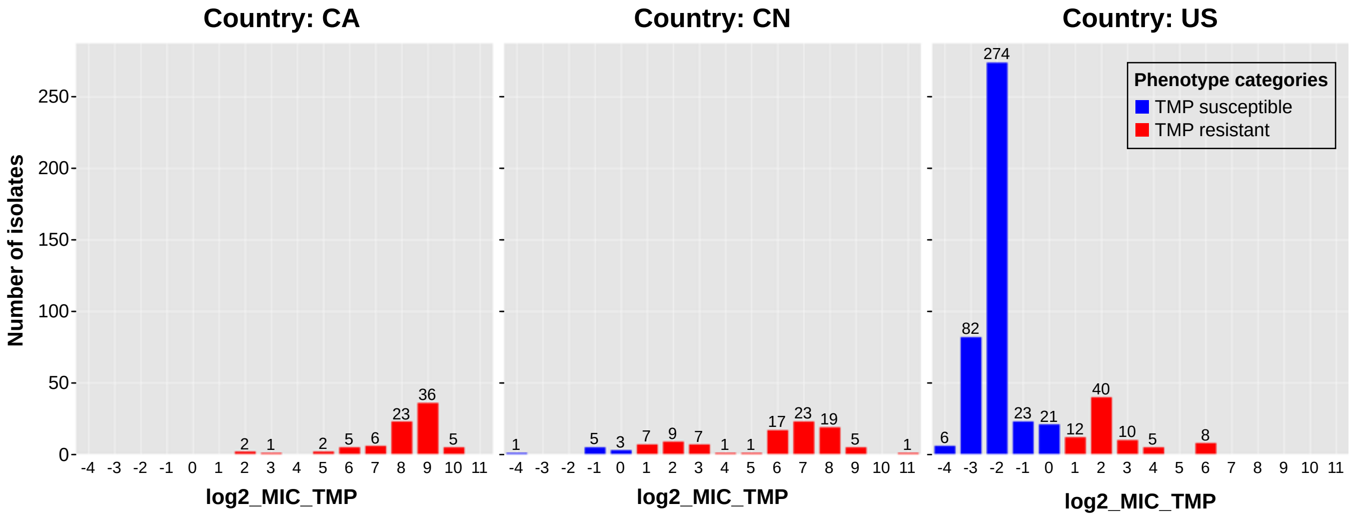

Fig. S1. Distribution of samples by country. The number of isolates is plotted by Log_2 MIC and colored by phenotypes (blue: susceptible, red: resistant); isolates are considered susceptible to TMP if having Log_2 MIC ≤ 0 (*i.e.* TMP MIC ≤ 1 μg/ml) and resistant if having Log_2 MIC ≥ 1 (*i.e.* TMP MIC ≥ 2 μg/ml). CA: Canada, CN: China, US: United States.

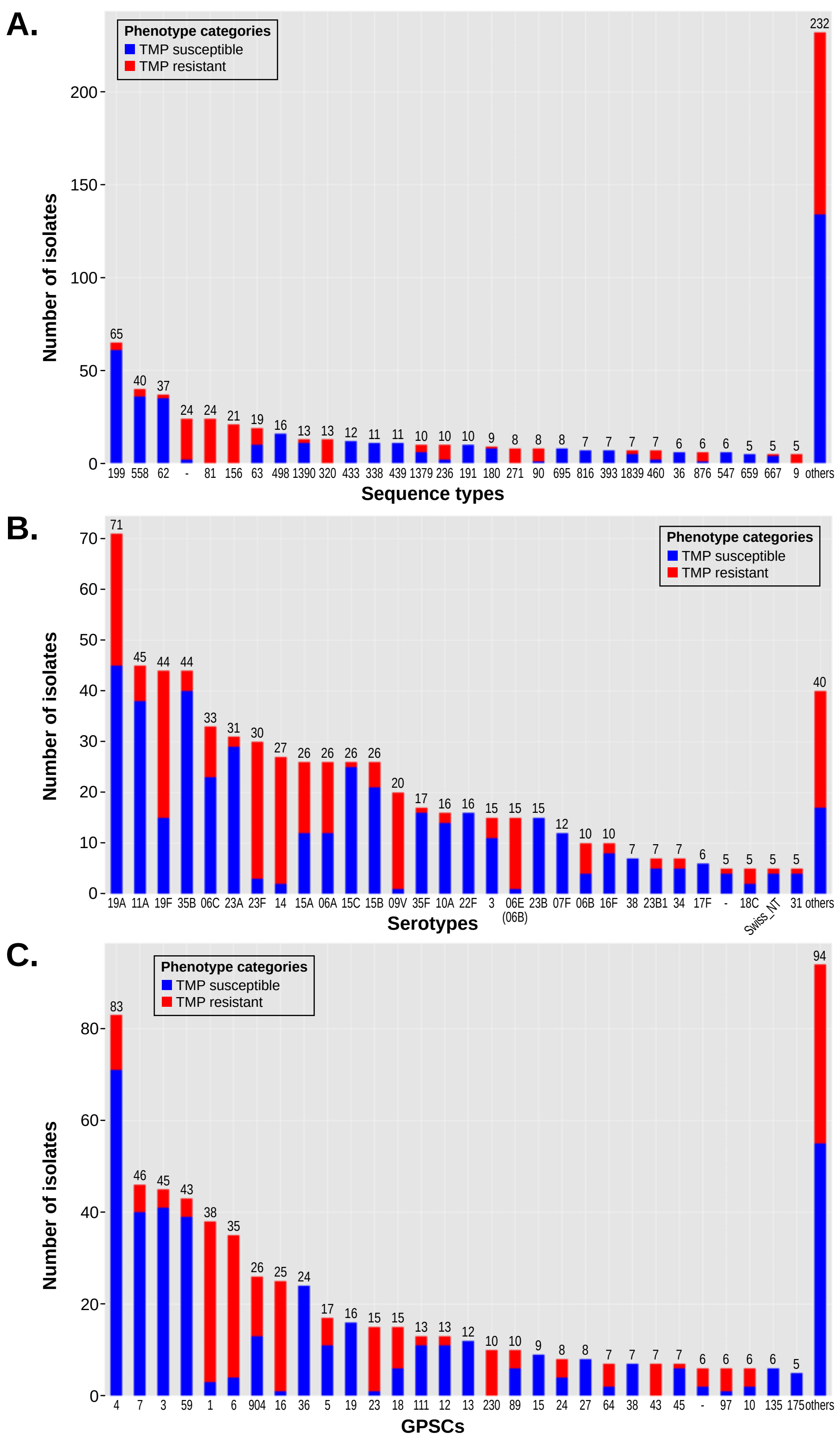

Fig. S2. Distribution of samples by (A) sequence types (STs), (B) serotypes and (C) global pneumococcal sequence clusters (GPSCs). The blue and red parts indicate the proportion of susceptible (Log$_2$ MIC ≤ 0 *i.e.* TMP MIC ≤ 1 μg/ml) and resistant (Log$_2$ MIC ≥ 1 *i.e.* TMP MIC ≥ 2 μg/ml) isolates, respectively. -, unassigned STs / serotypes / GPSCs.

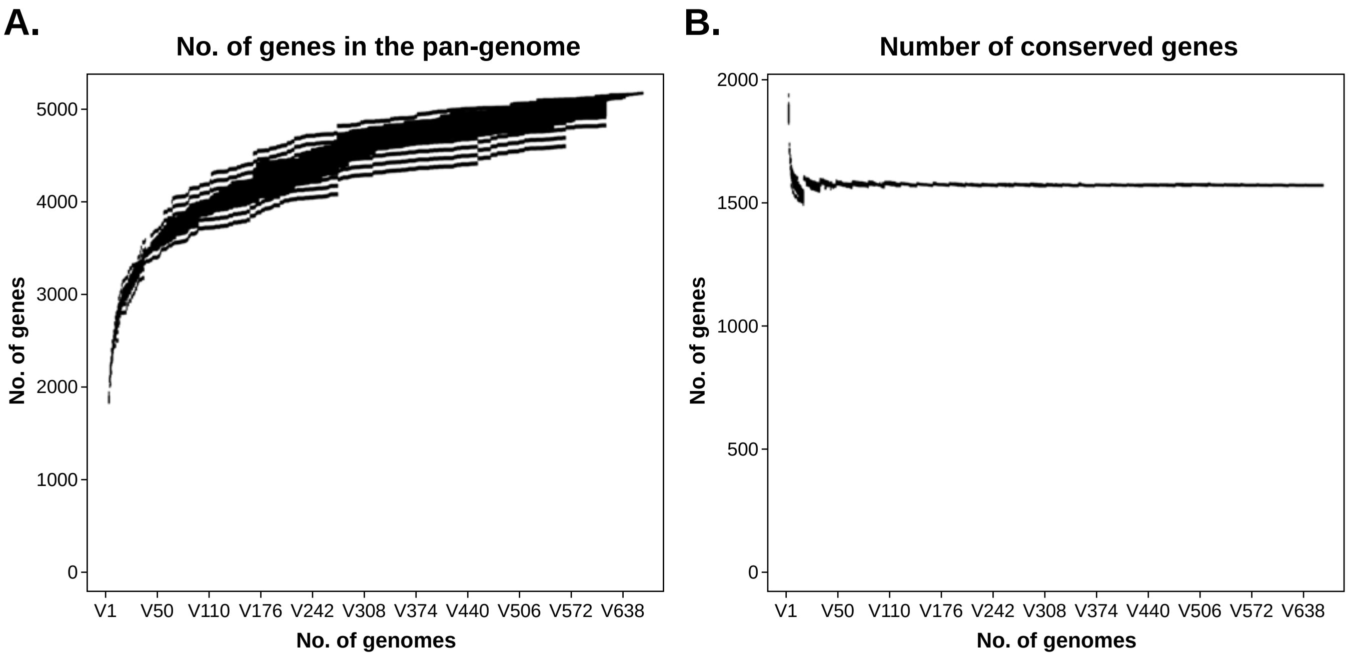

Fig. S3. The number of genes in the pangenome (A) and the core genome (B) are plotted as a function of the number of genomes added.

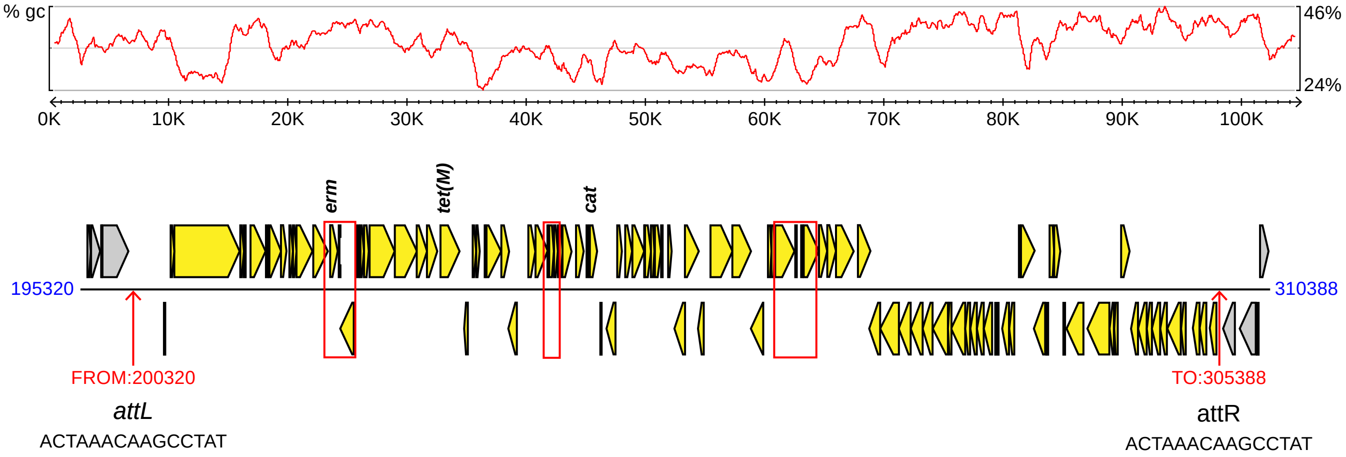

Fig. S4. Gene map of a putative ICE in *S. pneumoniae* CCRI1386 with its attachment sites (*attL* and *attR*). AMR genes are annotated above the gene track. The COGs associated with TMP resistance are highlighted with red boxes. See Table S2A for more details.

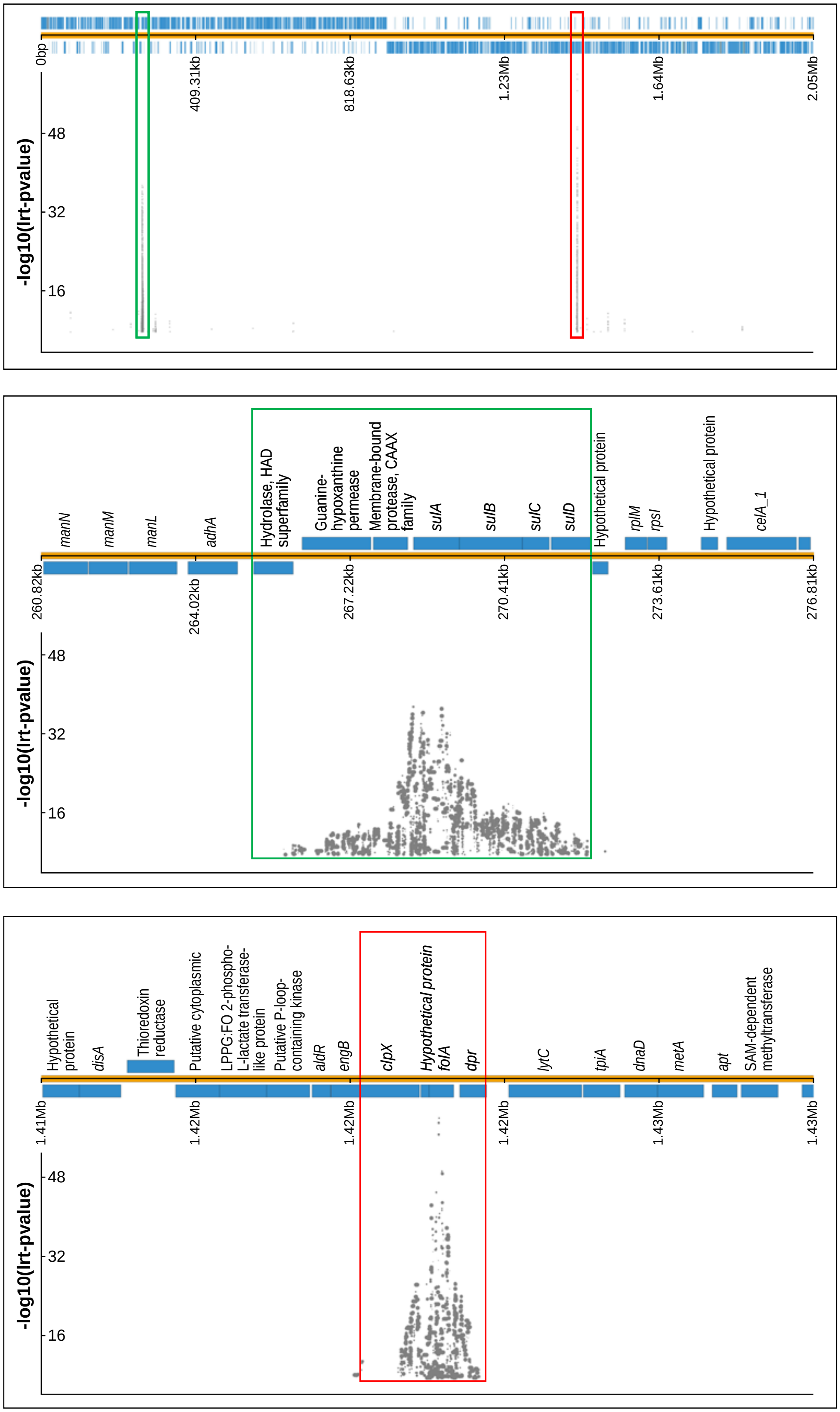

Fig. S5. Manhattan plot of significant k-mers associated with TMP resistance. The *sulA* locus is highlighted by a green box in the top panel and expanded in the second panel. The *folA* locus is highlighted by a red box in the top panel and expanded in the third panel.

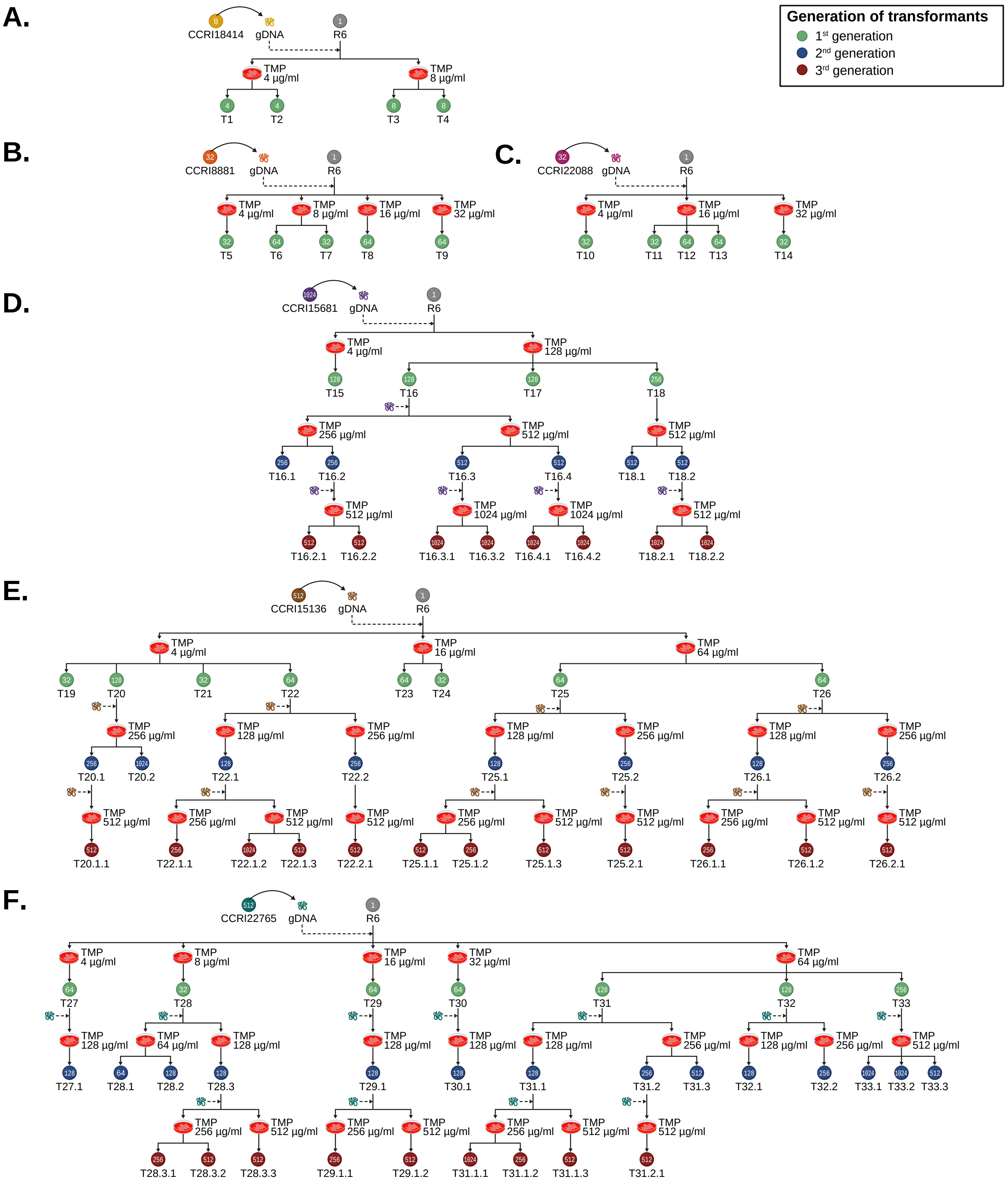

Fig. S6. Whole genome transformation and TMP resistance reconstruction of *S. pneumoniae* CCRI18414 (A), CCRI8881 (B), CCRI22088 (C), CCRI15681 (D), CCRI15136 (E) and CCRI22765 (F). Green, blue and magenta circles represent transformants obtained at each transformation steps, according to the coloring scheme shown as inset. The TMP concentration used for selecting the transformants on plates (red petri dishes) is shown next to plates. TMP MICs (µg/ml) of each transformant are indicated in the circles.

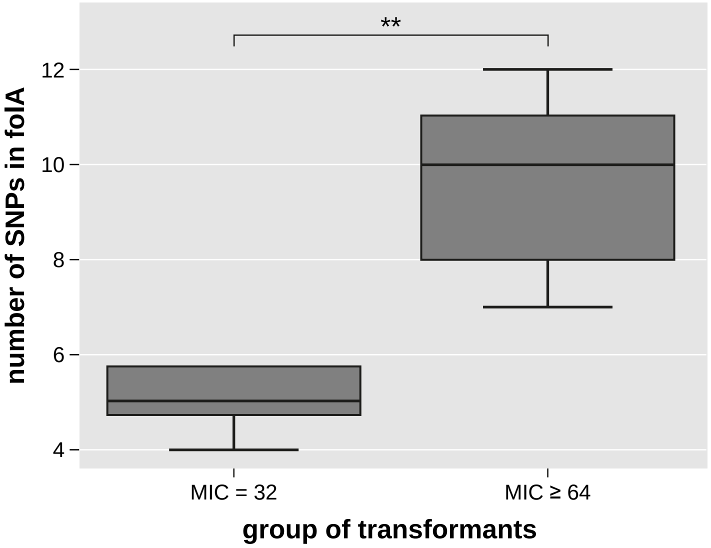

Fig. S7. Comparison of the number of SNPs in *folA* transferred in transformants with a TMP MIC of 32 µg/ml *versus* those with a MIC ≥ 64 µg/ml in the first round of transformation of *S. pneumoniae* R6 with gDNAs derived from three highly TMP resistant strains (CCRI15681, CCRI15136 and CCRI22765). The two groups were compared using the Mann-Whitney test, **, $p < 0.01$.

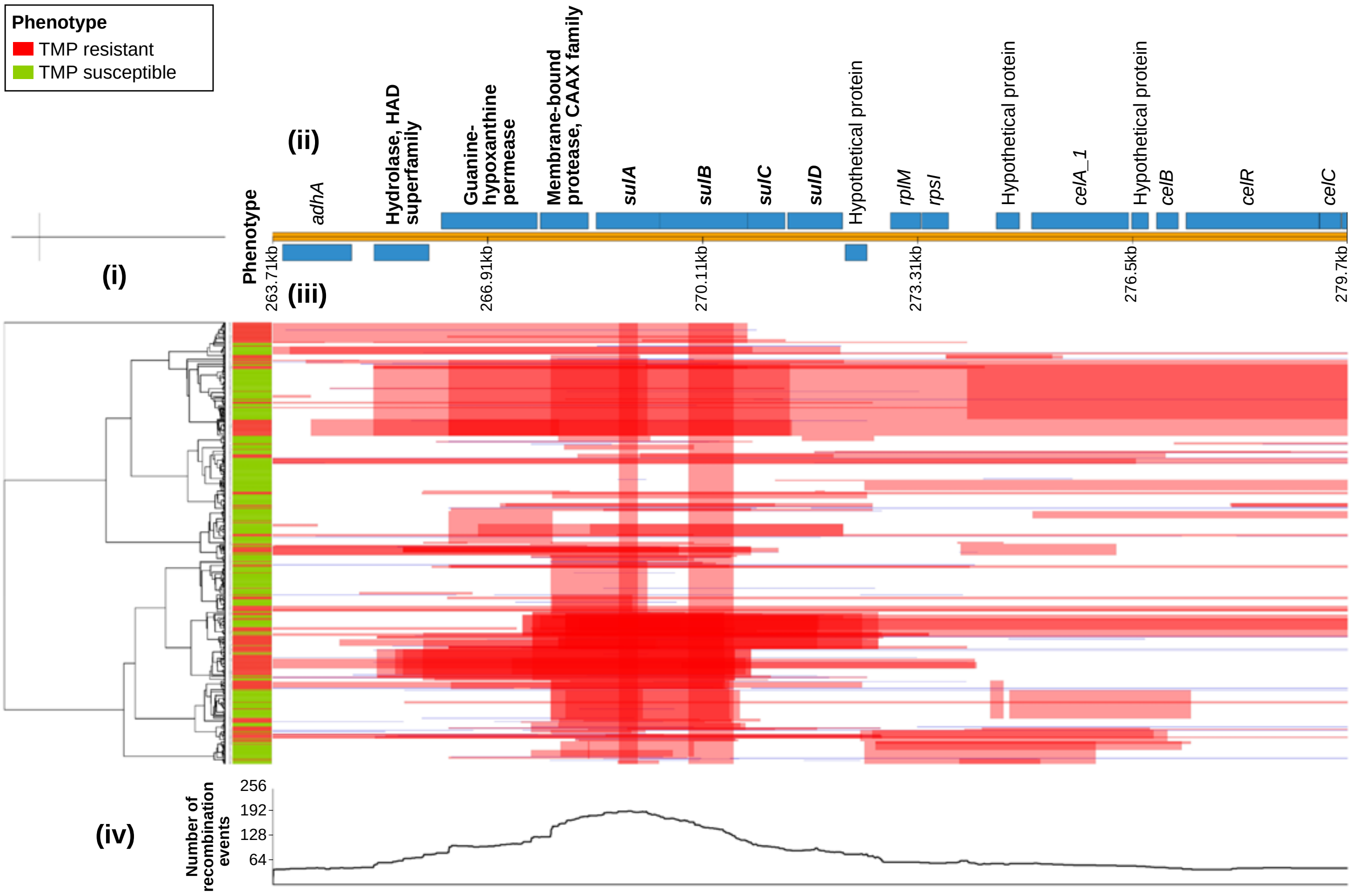

Fig. S8. Recombination inferred by Gubbins, focused on the *sulA* locus. (i) Recombination-free maximum-likelihood tree of the 662 *S. pneumoniae* strains used in this study. The color strip represents the phenotype of each strains (red, TMP resistant; green, TMP susceptible). (ii) Gene map of the *S. pneumoniae* D39V reference genome. Genes from the *sulA* locus are highlighted in bold. (iii) Recombination blocks spanning the taxa in which they are detected and the genes affected. Red blocks affect n > 1 strains, blue blocks affect n = 1 strain. Overlapping blocks increase the density of the colour. (iv) The number of recombination events is plotted by position in the reference genome.

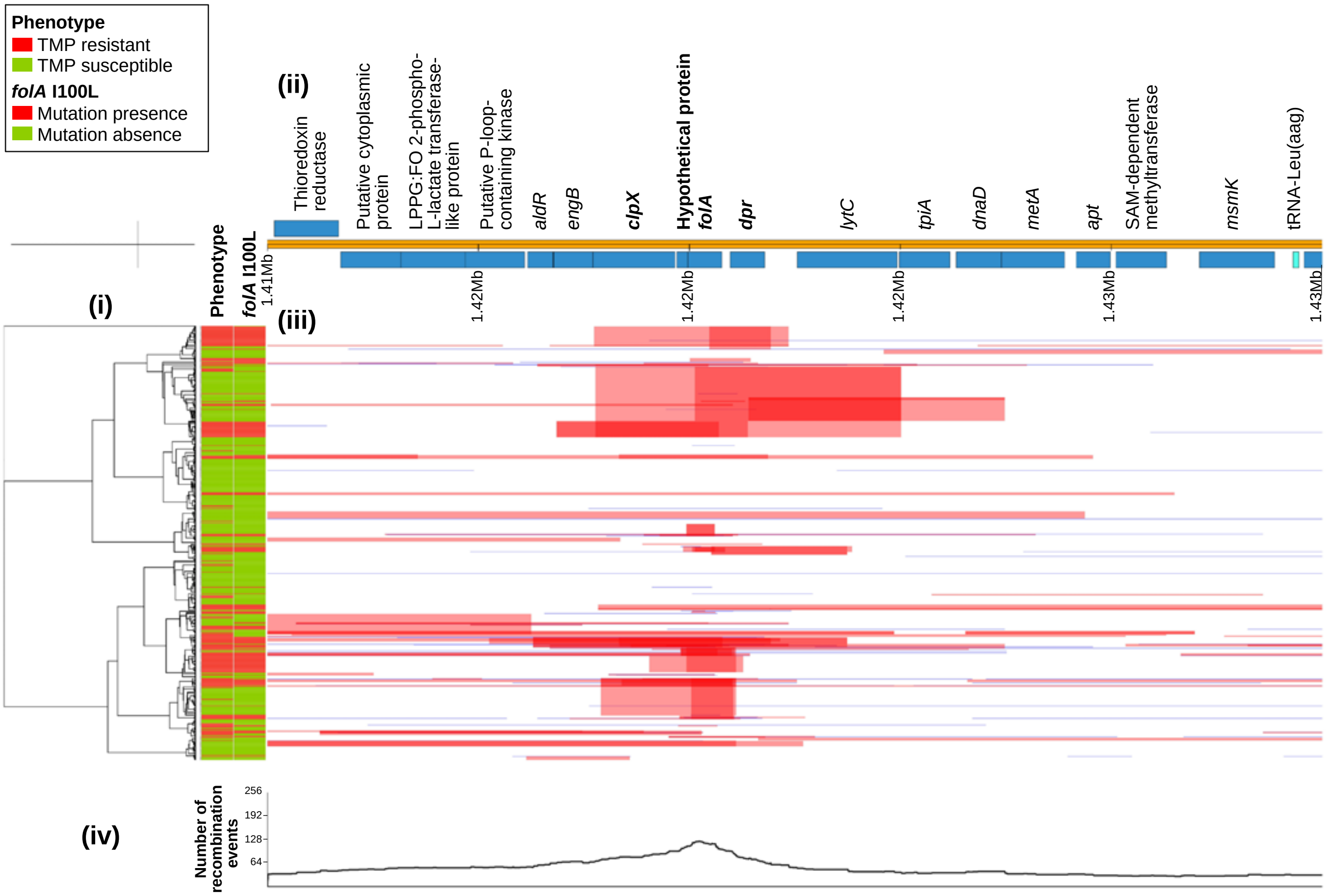

Fig. S9. Recombination inferred by Gubbins focused on the *folA* locus, display as described in Fig. S8. In panel (ii), the genes from the *folA* locus are highlighted in bold.

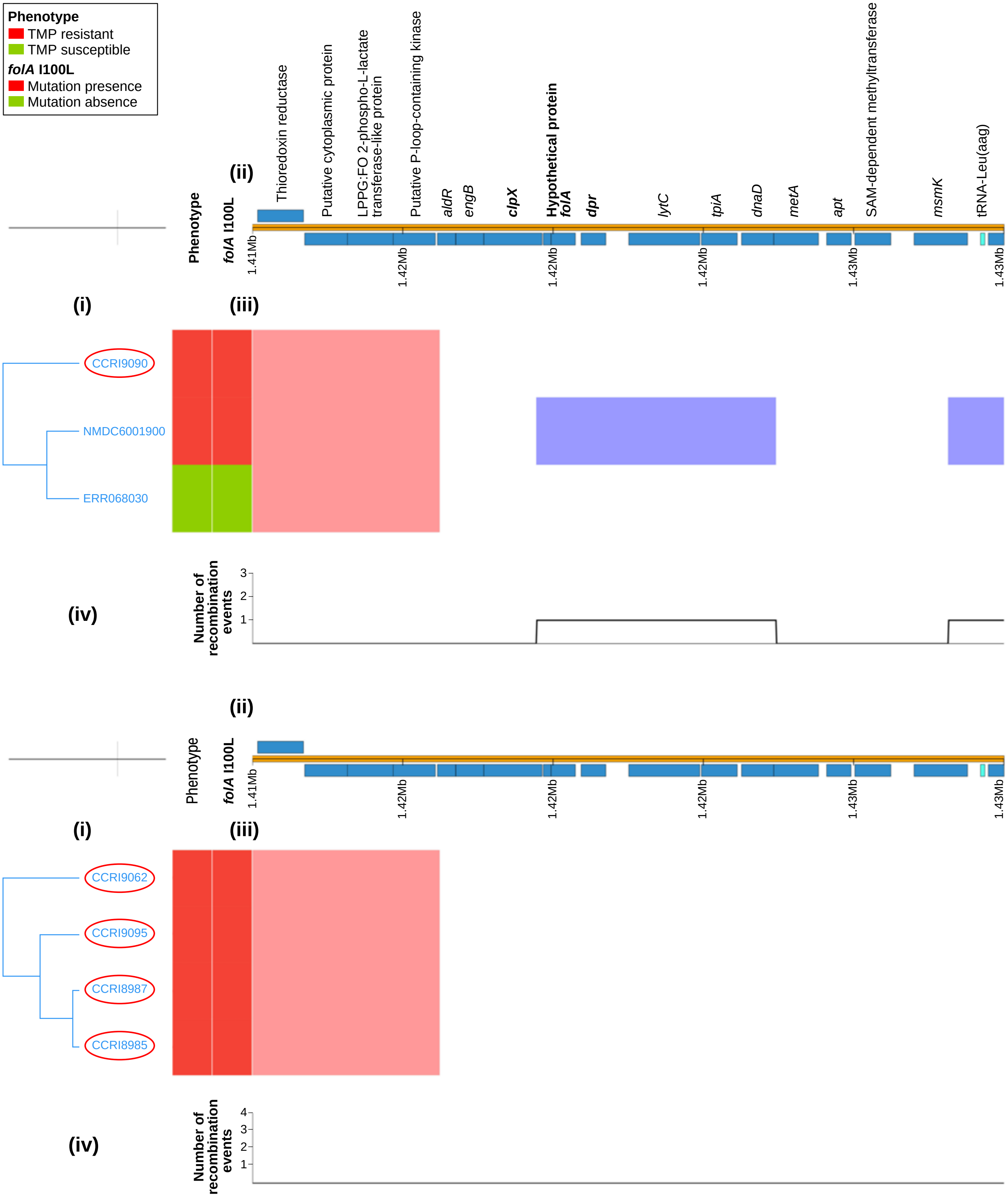

Fig. S10. Zoom-in of Fig. S9 on lineages with no recombination block at the *folA* locus. These strains are highlighted by red circles in panel (i).

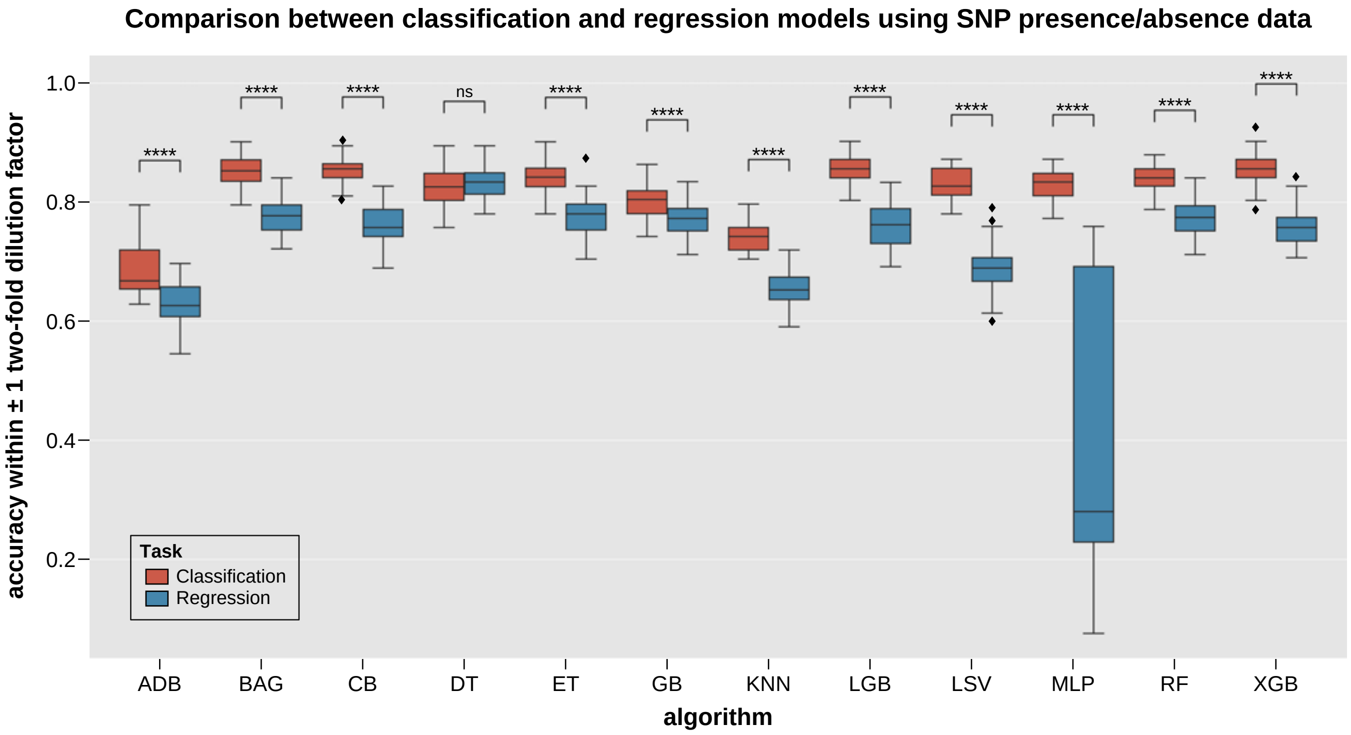

Fig. S11. Comparison of performance based on the accuracy within ± 1 two-fold dilution factor between classification (red) and regression (blue) models using SNP presence/absence data. Models were compared using Welch's t-test with Bonferroni correction for multiple testing (****$p < 0.0001$, ***$p < 0.001$, **$p < 0.01$, *$p < 0.05$, ns: non-significant). ADB: AdaBoost, BAG: Bagging, CB: CatBoost, DT: Decision Tree, ET: Extra-trees, GB: Gradient Boosting, KNN: k-nearest neighbors, LGB: LightGBM, LSV: Linear Support Vector, MLP: Multi-layer Perceptron, RF: Random Forest, XGB: XGBoost.

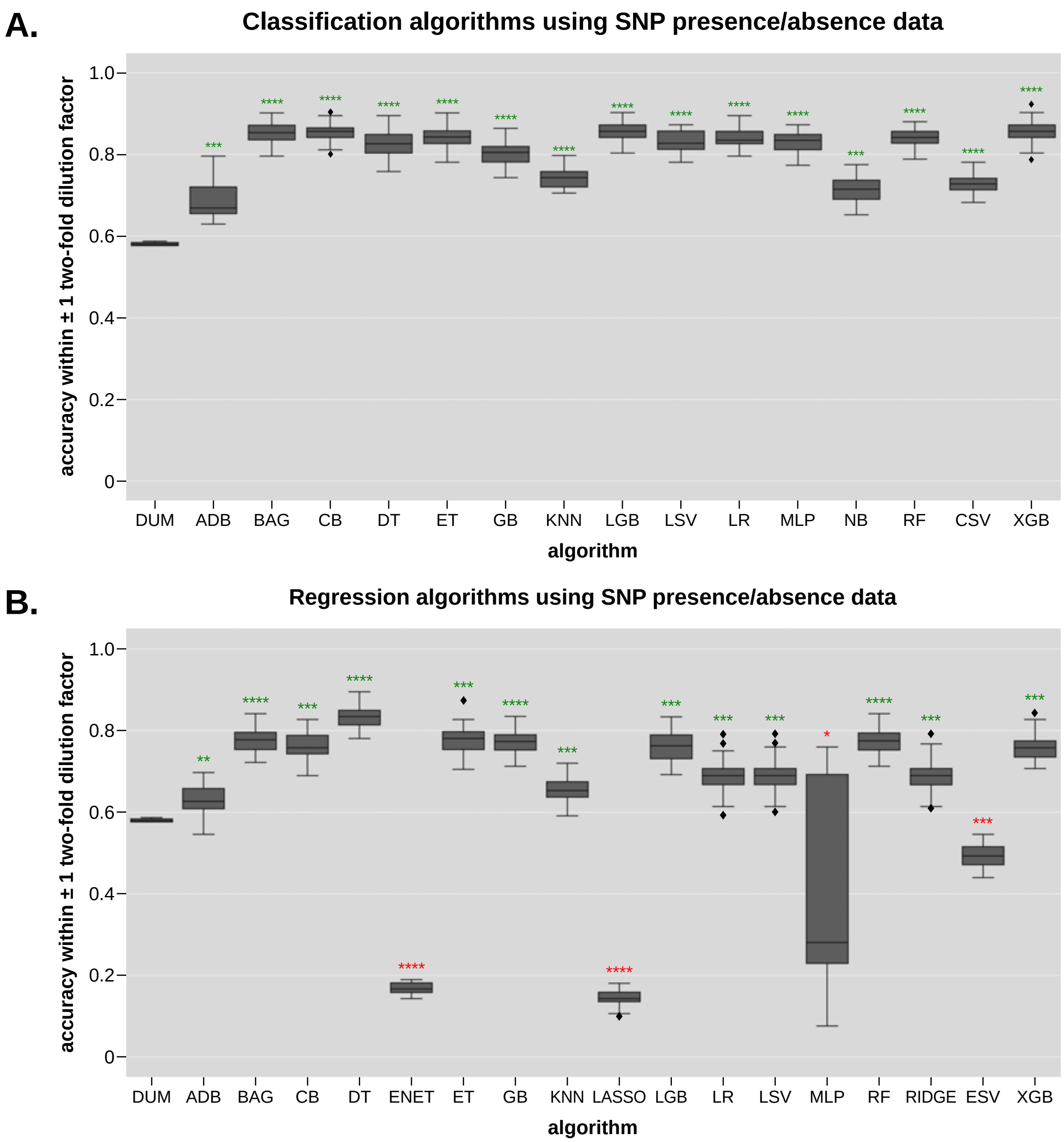

Fig. S12. Comparison of performance based on the accuracy within ± 1 two-fold dilution factor between machine learning models using the SNP presence/absence data as input and the baseline (dummy model). Models were compared using Welch's t-test with Bonferroni correction for multiple testing (****p < 1E-40, ***p < 1E-20, **p < 1E-10, *p < 0.05). (A) Classification models. Dummy model used the “most_frequent” strategy (*i.e.* always predicts the most frequent class in the training set). DUM: Dummy, ADB: AdaBoost, BAG: Bagging, CB: CatBoost, DT: Decision Tree, ET: Extra-trees, GB: Gradient Boosting, KNN: k-nearest neighbors, LGB: LightGBM, LSV: Linear Support Vector, LR: Logistic Regression, MLP: Multi-layer Perceptron, NB: Naïve Bayes, RF: Random Forest, CSV: C-Support Vector, XGB: XGBoost. (B) Regression models. Dummy model used the “median” strategy (*i.e.* always predicts the median of the training set). DUM: Dummy, ADB: AdaBoost, BAG: Bagging, CB: CatBoost, DT: Decision Tree, ENET: ElasticNet Regression, ET: Extra-trees, GB: Gradient Boosting, KNN: k-nearest neighbors, LASSO: Lasso Regression, LGB: LightGBM, LR: Linear Regression, LSV: Linear Support Vector, MLP: Multi-layer Perceptron, RF: Random Forest, RIDGE: Ridge regression, ESV: Epsilon-Support Vector, XGB: XGBoost.

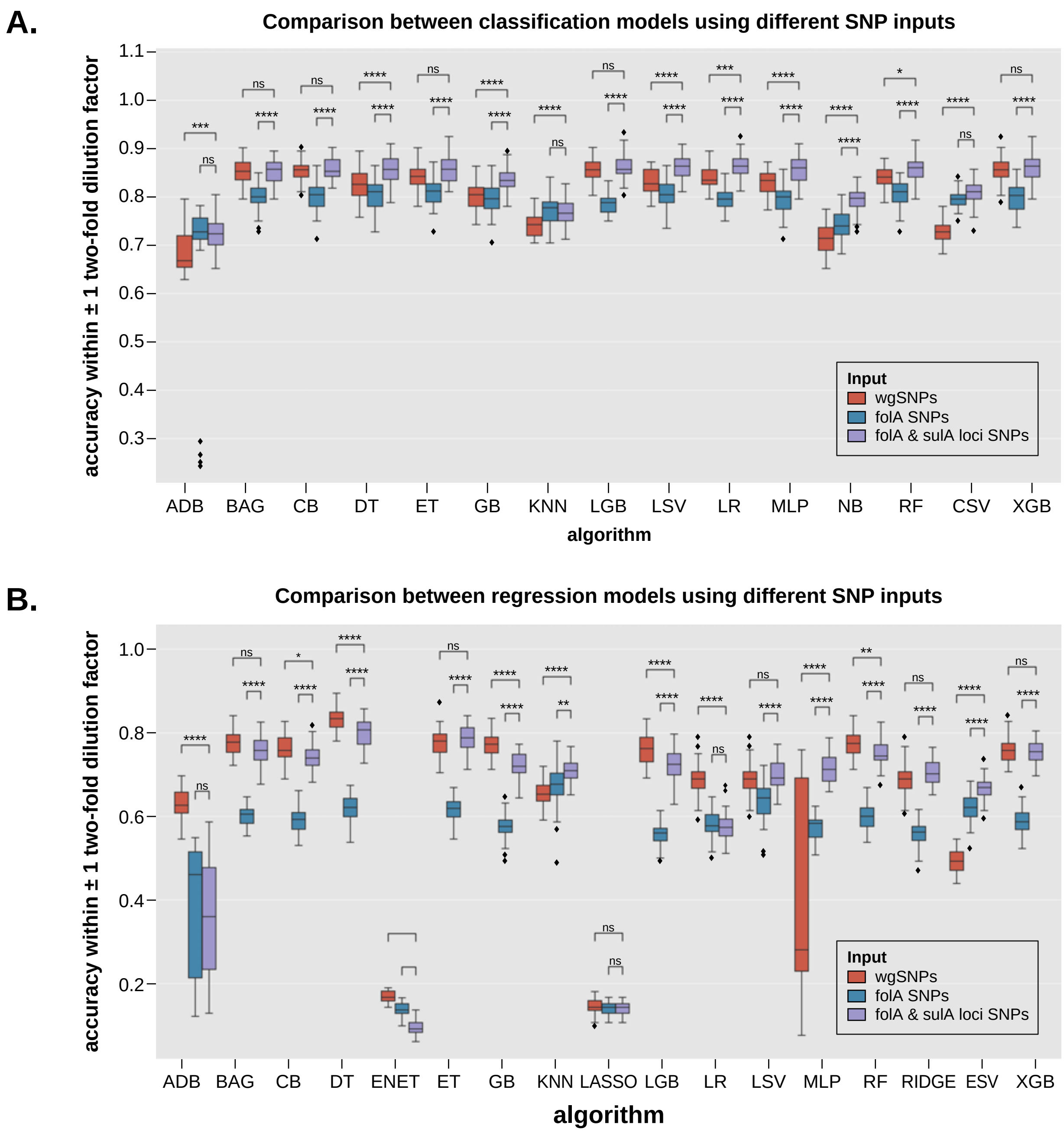

Fig. S13. Comparison of performance based on the accuracy within ± 1 two-fold dilution factor between the same algorithm using different inputs data (whole-genome SNPs: red, *folA* SNPs: blue, *folA* and *sulA* loci SNPs: purple). Models were compared using Welch's t-test with Bonferroni correction for multiple testing (****$p < 0.0001$, ***$p < 0.001$, **$p < 0.01$, *$p < 0.05$, ns: non-significant). (A) Classification models. ADB: AdaBoost, BAG: Bagging, CB: CatBoost, DT: Decision Tree, ET: Extra-trees, GB: Gradient Boosting, KNN: k-nearest neighbors, LGB: LightGBM, LSV: Linear Support Vector, LR: Logistic Regression, MLP: Multi-layer Perceptron, NB: Naïve Bayes, RF: Random Forest, CSV: C-Support Vector, XGB: XGBoost. (B) Regression models. ADB: AdaBoost, BAG: Bagging, CB: CatBoost, DT: Decision Tree, ENET: ElasticNet Regression, ET: Extra-trees, GB: Gradient Boosting, KNN: k-nearest neighbors, LASSO: Lasso Regression, LGB: LightGBM, LR: Linear Regression, LSV: Linear Support Vector, MLP: Multi-layer Perceptron, RF: Random Forest, RIDGE: Ridge regression, ESV: Epsilon-Support Vector, XGB: XGBoost.

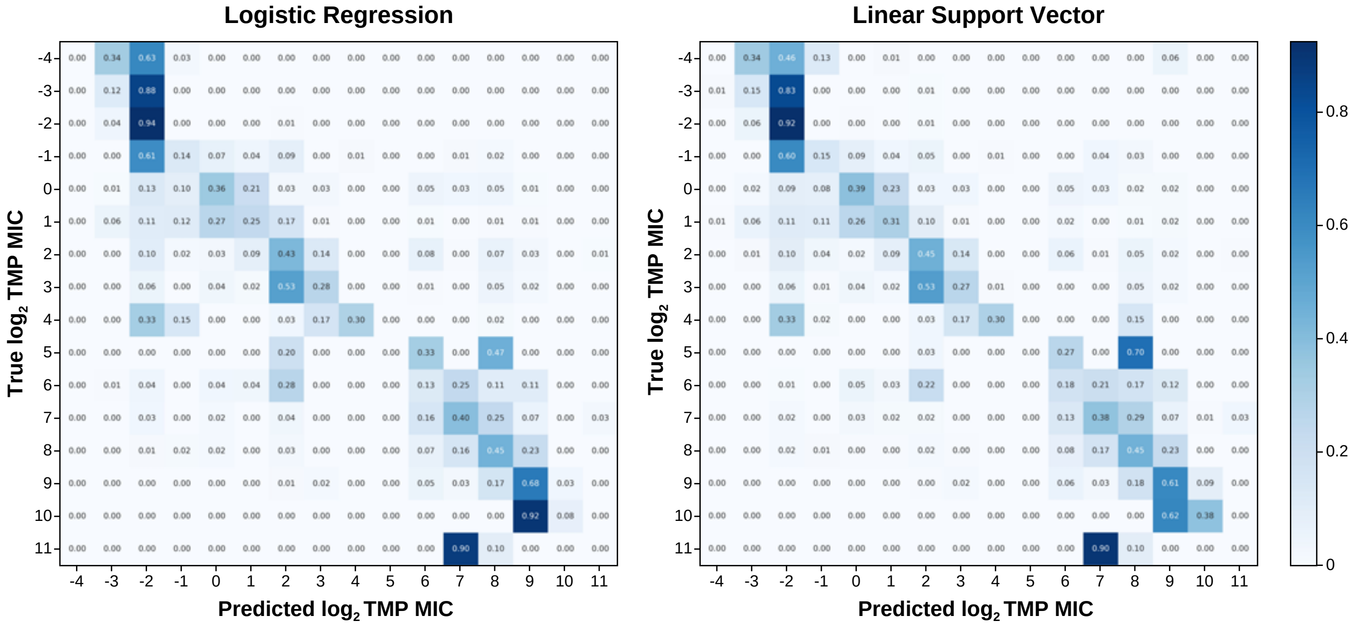

Fig. S14. Confusion matrix for the TMP MIC predicted by the Logistic Regression classification (left) and Linear Support Vector (right) models based on the *folA* and *sulA* loci as a function of the true TMP MIC. The matrix is normalized over the true Log_2 TMP MIC (rows), *i.e.* the sum of each row is 1.

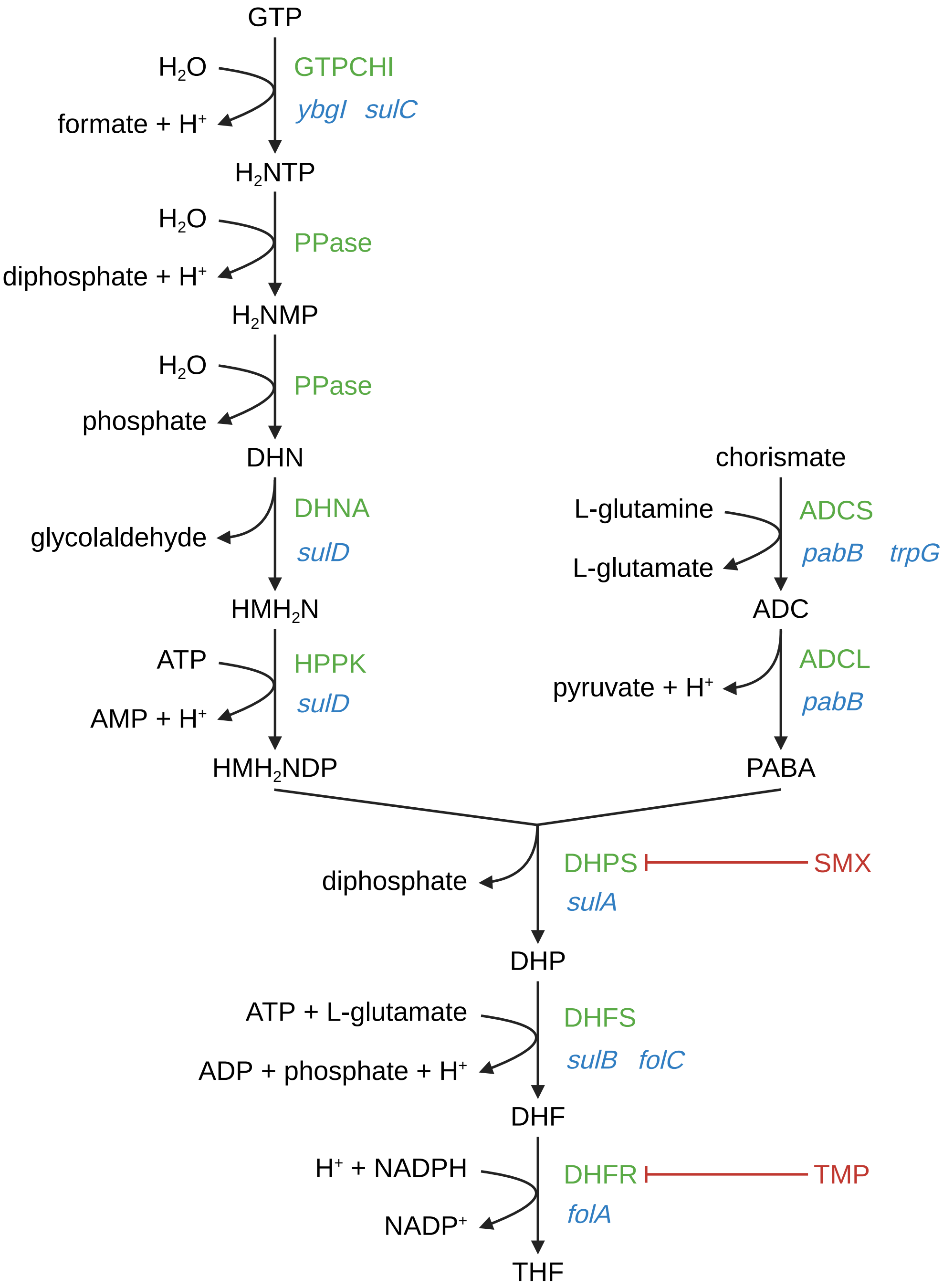

Fig. S15. Tetrahydrofolate biosynthesis pathway in *S. pneumoniae*. The enzymes (in green) catalyzing each reaction and their corresponding genes (in blue) are indicated. In red are indicated the enzymatic steps targeted by trimethoprim (TMP) and sulfamethoxazole (SMX). Metabolites: GTP, Guanosine-5'-triphosphate; H_2NTP, 7,8-dihydroneopterin 3'-triphosphate; H_2NMP, 7,8-dihydroneopterin 3'-phosphate; DHN, D-erythro-7,8-dihydroneopterin; HMH_2N, 6-(hydroxymethyl)-7,8-dihydropterin; HMH_2NDP, (7,8-dihy-dropterin-6-yl)methyl diphosphate; ADC, 4-amino-4-deoxychorismate; PABA, 4-aminobenzoate; DHP, 7,8-dihydropteroate; DHF, 7,8-dihydrofolate; THF, tetrahydrofolate. Enzymes: GTPCHI, GTP cyclohy-drolase I; PPase, phosphatase; DHNA, dihydroneopterin aldolase; HPPK, 2-amino-4-hydroxy-6-hydroxy-methyldihydropterin pirophosphokinase; ADCS, aminodeoxychorismate synthase; ADCL, 4-amino-4-deoxychorismate lyase; DHPS, dihydropteroate synthase; DHFS, dihydrofolate synthase; DHFR, dihydrofolate reductase.

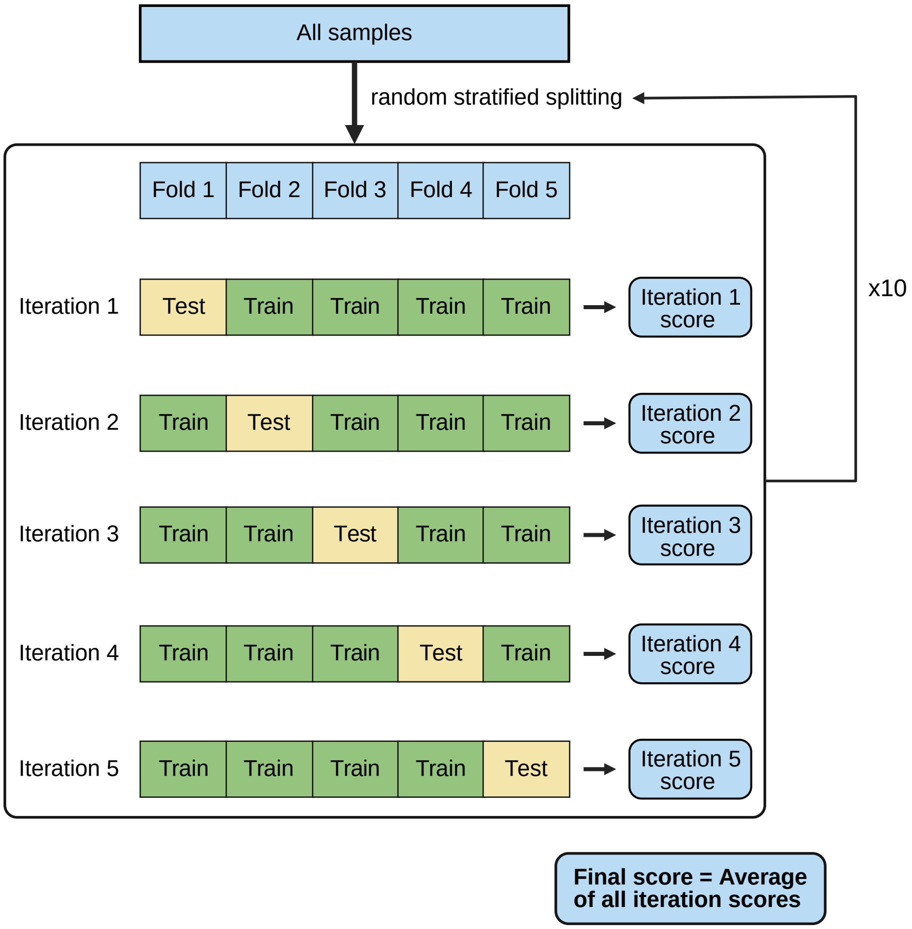

Fig. S16. Cross-validation (CV) scheme. A 10-times-repeated stratified 5-fold CV was applied. The 5-fold CV works by splitting the data into 5 folds. These folds are iterated through 5 times. During each iteration, 4 of the 5 folds are used as the training set, and the remaining 5th fold is used as the validation set. The performance metrics are measured after each iteration. The above procedure is repeated 10 times.

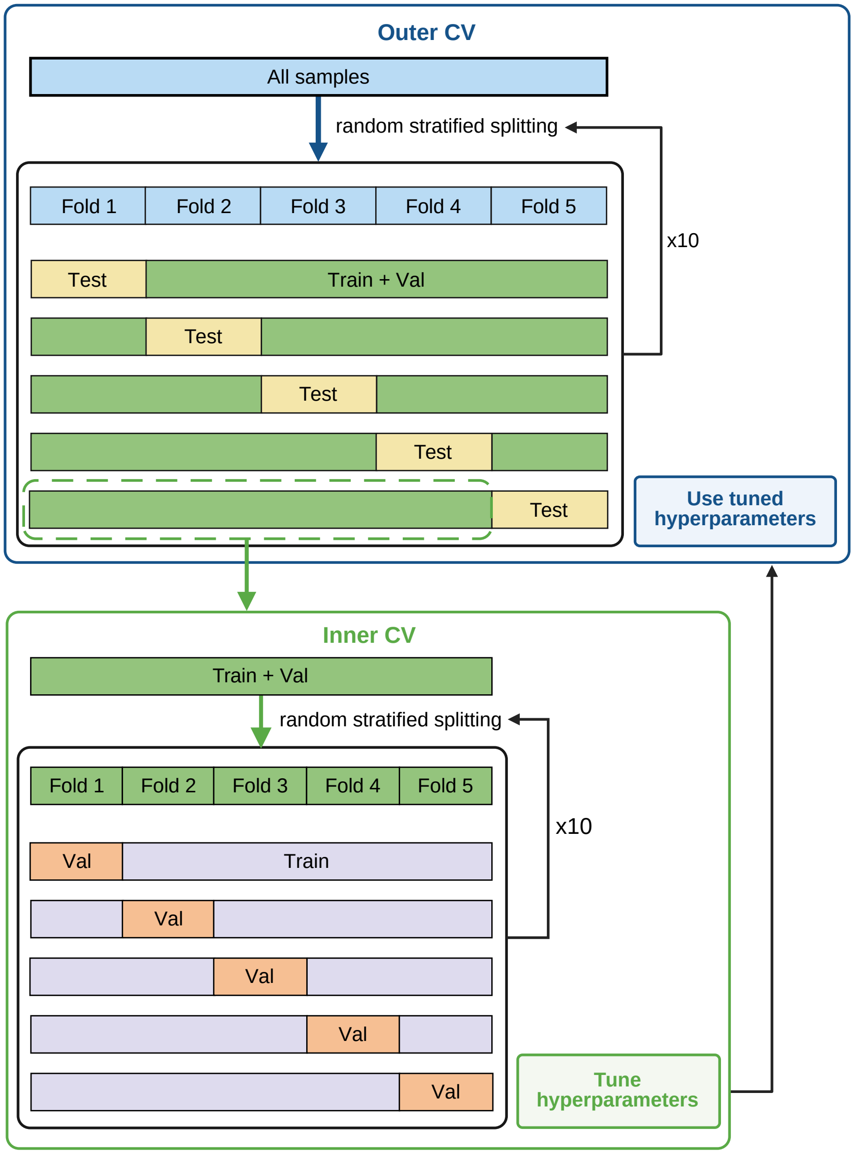

Fig. S17. Nested cross-validation (CV) scheme. It involves two CV loops nested within each other, each one is a 10-times-repeated stratified 5-fold CV. In the outer loop, different subsets of the data are selected as training and test sets for model evaluation. Within each iteration of the outer loop, the inner loop is used to tune hyperparameters using the training set, then the tuned hyperparameters are evaluated on the test set. This procedure is repeated for each iteration of the outer loop.
